# MTGR1 is required to maintain small intestinal stem cell populations

**DOI:** 10.1038/s41418-024-01346-x

**Published:** 2024-07-25

**Authors:** Sarah P. Short, Rachel E. Brown, Zhengyi Chen, Jennifer M. Pilat, Bailey A. McElligott, Leslie M. Meenderink, Alexander C. Bickart, Koral M. Blunt, Justin Jacobse, Jing Wang, Alan J. Simmons, Yanwen Xu, Yilin Yang, Bobak Parang, Yash A. Choksi, Jeremy A. Goettel, Ken S. Lau, Scott W. Hiebert, Christopher S. Williams

**Affiliations:** 1 Department of Medicine, Vanderbilt University Medical Center, Nashville, TN USA; 2 Program in Cancer Biology, Vanderbilt University, Nashville, TN USA; 3 Department of Internal Medicine, University of Iowa, Iowa City, IA USA; 4 Vanderbilt University School of Medicine, Vanderbilt University, Nashville, TN USA; 5 Program in Chemical and Physical Biology, Vanderbilt University, Nashville, TN USA; 6 Epithelial Biology Center, Vanderbilt University Medical Center, Nashville, TN USA; 7 Veterans Affairs Tennessee Valley Health Care System, Nashville, TN 37232 USA; 8 The Ohio State University College of Medicine, Columbus, OH USA; 9 Willem-Alexander Children’s Hospital, Department of Pediatrics, Leiden University Medical Center, Leiden, The Netherlands; 10 Department of Pathology, Microbiology, and Immunology, Vanderbilt University Medical Center, Nashville, TN USA; 11 Department of Biostatistics, Vanderbilt University, Nashville, TN USA; 12 Center for Quantitative Sciences, Vanderbilt University Medical Center, Nashville, TN USA; 13 Department of Cell and Developmental Biology, Vanderbilt University, Nashville, TN USA; 14 Department of Medicine, Weill Cornell Medicine, New York, NY 10021 USA; 15 Department of Biochemistry, Vanderbilt University, Nashville, TN USA

**Keywords:** Epigenetics, Stem-cell research

## Abstract

Undifferentiated intestinal stem cells (ISCs) are crucial for maintaining homeostasis and resolving injury. *Lgr5*+ cells in the crypt base constantly divide, pushing daughter cells upward along the crypt axis where they differentiate into specialized cell types. Coordinated execution of complex transcriptional programs is necessary to allow for the maintenance of undifferentiated stem cells while permitting differentiation of the wide array of intestinal cells necessary for homeostasis. Previously, members of the myeloid translocation gene (MTG) family have been identified as transcriptional co-repressors that regulate stem cell maintenance and differentiation programs in multiple organ systems, including the intestine. One MTG family member, myeloid translocation gene related 1 (MTGR1), has been recognized as a crucial regulator of secretory cell differentiation and response to injury. However, whether MTGR1 contributes to the function of ISCs has not yet been examined. Here, using *Mtgr1*^*−/−*^ mice, we have assessed the effects of MTGR1 loss specifically in ISC biology. Interestingly, loss of MTGR1 increased the total number of cells expressing *Lgr5*, the canonical marker of cycling ISCs, suggesting higher overall stem cell numbers. However, expanded transcriptomic and functional analyses revealed deficiencies in *Mtgr1*-null ISCs, including deregulated ISC-associated transcriptional programs. Ex vivo, intestinal organoids established from *Mtgr1*-null mice were unable to survive and expand due to aberrant differentiation and loss of stem and proliferative cells. Together, these results indicate that the role of MTGR1 in intestinal differentiation is likely stem cell intrinsic and identify a novel role for MTGR1 in maintaining ISC function.

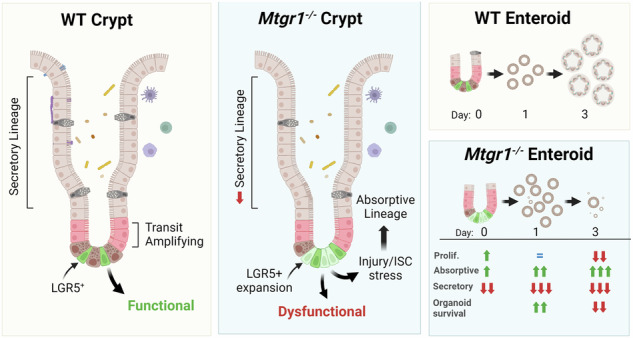

## Introduction

The intestinal epithelium is under constant metabolic, mechanical, and microbial stress and is thus in a continual state of regeneration and renewal. While models for cell function in the gut are evolving, it has been repeatedly demonstrated that intestinal stem cells (ISCs) are key to maintaining intestinal homeostasis and response to injury [1]. Maintenance of intestinal health and regeneration requires the orchestrated execution of stem cell, early progenitor, and differentiation programs, usually via coordinated activation and suppression of transcriptional circuits, to balance stem cell function with downstream lineage allocation [2].

To accommodate the constant cellular turnover of the intestinal epithelium, ISCs rapidly proliferate in the crypt base, pushing their daughter cells out of the crypt and up the intestinal villus while undergoing terminal differentiation into an assortment of secretory and absorptive cell types. These highly proliferative crypt base ISCs, termed crypt base columnar cells (CBCs), are identified by their expression of the leucine-rich repeat containing G-protein coupled receptor 5 (*Lgr5*) gene [1]. *Lgr5* expression is highly regulated, most notably by Wnt and Notch signaling pathways, and is suppressed upon exit of the stem cell niche and activation of cellular differentiation programs [3]. As proliferation continues, daughter cells from CBCs move further up the crypt base into the transit amplifying (TA) compartment. Here, cells proliferate even more rapidly than parental CBCs, yet simultaneously begin lineage commitment and terminal differentiation.

In addition to *Lgr5* + CBCs, other populations of ISCs have been identified, many of which are less proliferative than CBCs at homeostasis but are “activated” as part of intestinal regenerative programs. These cell populations, denoted by expression of specific genes such as *Bmi1*, *mTert*, *Hopx*, *Lrig1*, and *Clu*, often reside higher in the crypt base than CBCs in the +4/+5 position and may have begun the process of lineage commitment [4, 5]. However, functional compensation by these slowly cycling ISC populations may still require dedifferentiation and interconversion to CBCs, in which ISCs gain expression of *Lgr5* and take up residence in the crypt base. Interestingly, the ability to reconstitute the CBC population has even been noted in committed progenitors of the secretory and absorptive lineages [6–9]. Thus, coordinating ISC function and differentiation among differentiated cell types and different ISC populations is a far from linear process, yet remains a crucial component of both intestinal homeostasis and injury responses.

MTGR1 (*Cbfa2t2*) is a member of the three-protein myeloid translocation gene (MTG) family of transcriptional co-repressors, which were originally identified in translocation fusion proteins driving acute myeloid leukemia [10]. MTGs, also including MTG16 and MTG8, serve as scaffolding proteins that orchestrate the formation of repression complexes containing histone deacetylases, other co-repressor proteins, and DNA binding factors, thereby modifying the chromatin at key loci [11]. In the intestine, MTGs serve important functions, as MTG-deficient mice display a range of unprovoked intestinal phenotypes. For example, loss of MTG8 (*Mtg8*^*−/−*^) resulted in deletion of the midgut, while loss of MTG16 or MTGR1 (*Mtg16*^*−/−*^ and *Mtgr1*^*−/−*^) altered intestinal proliferation, apoptosis, migration, and lineage specification [12–15]. *Mtgr1*^*−/−*^ mice are also exquisitely sensitive to dextran sodium sulfate (DSS)-induced injury, with marked depletion of viable, regenerative crypts post-injury [16]. Finally, we have previously identified MTGR1 as a key regulator of intestinal differentiation into the secretory cell fate, as *Mtgr1*^*−/−*^ mice have greatly reduced numbers of Paneth, goblet, and enteroendocrine cells [14, 17].

Our previous work has uncovered roles for MTGs in modulating Wnt and Notch signaling pathways [17, 18]. Despite contributions to these key ISC-associated signaling pathways, the exact role of MTGR1 in ISC biology remains incompletely understood. Here, we have assessed the impact of *Mtgr1* deficiency in the small intestine both in vivo and ex vivo. Together, these studies uncover a crucial role for MTGR1 in maintaining proper ISC function and regulating absorptive differentiation thus expanding our knowledge of the mechanisms regulating intestinal differentiation and regeneration.

## Methods and materials

### Mouse models

*Mtgr1*^*−/−*^ mice were previously established and characterized [14]. *Lgr5-EGFP-IRES-creERT2* mice were a generous gift from Dr. Robert Coffey [1]. Male and female age-matched, littermate WT and *Mtgr1*^*−/−*^ mice were used for all experiments. Mice were cohoused and maintained on standard chow with 12-hour light/dark cycles. All in vivo procedures were carried out in accordance with protocols approved by the Vanderbilt Institutional Animal Care and Use Committee.

### Enteroid culture

3D small intestinal organoids, or enteroids, were established from duodenal crypts isolated from 8–12-week old WT and *Mtgr1*^*−/−*^ mice as previously described ([17, 19], and Supplementary Methods). To determine plating efficiency, the number of viable enteroids was assessed at day 1 post-plating and normalized to the number of crypts plated. Viability was determined by daily enteroid counts per 12-well Matrigel patty and normalized to the number of enteroids established on day 1. ImageJ software (version 1.51) was used to measure enteroid size and count crypt buds. All plating experiments are representative of ≥2 independent experiments, with the total number of wells or enteroids noted in figure legends.

### Enteroid hMTGR1 addback

GFP and human *MTGR1* were cloned into the pLEX-307 vector (a gift from David Root, Addgene plasmid 41392). pLEX-307-GFP and pLEX-307-*MTGR1* were transfected into HEK 293T cells (ATCC CRL-3216) along with psPAX2 and pMD2.g (gifts from Didier Trono, Addgene plasmids 12260 and 12259). After 48 h, supernatants were collected and viral particles were concentrated by overnight centrifugation at 9500 × *g* at 4 °C. Pelleted lentiviral particles were resuspended in mouse Intesticult media (StemCell Technologies) supplemented with 10 µM Y-27632 (Tocris) and mixed with duodenal crypt isolations from WT or *Mtgr1*^*−/−*^ mice. Crypt/virus mixtures were incubated for 2 h at 37 °C prior to washing and plating in Matrigel plugs overlaid with ENR media supplemented with CHIR 99021 (3 µM, Tocris) and Y-27632. After 4 days, CHIR 99021 and Y-27632 were removed. Viability and gene expression were assessed at day 7 post-plating.

### Immunohistochemistry

Mice were sacrificed and intestinal tissue was “Swiss-rolled” prior to fixation in 10% neutral-buffered formalin. Intestinal samples were then paraffin-embedded and 5 µm sections were cut by the Vanderbilt Translational Pathology Shared Resource (TPSR). For enteroid staining, cultures were collected and fixed as described previously [20]. Samples were stained as we have done for prior studies [21, 22], with primary antibodies against E-cadherin (BD Biosciences, 1:500), Ki67 (Abcam, 1:1000), phospho-histone H3 (Millipore, 1:400), cleaved caspase-3 (Cell Signaling Technology, 1:400), or β-catenin (BD Biosciences, 1:500) and secondary antibodies conjugated to 488 or 594 Alexa Fluor dyes (Invitrogen). Nuclear staining was done with ProLong Gold antifade reagent with DAPI (Invitrogen). Staining was visualized with a Nikon Eclipse E800 microscope and Zyla SCMOS camera. Images were processed using Nikon NIS-Elements Basic Research software and quantified by blinded observer.

Tissue processing and brush border analysis was done as described previously [23, 24]. Duodenal segments were excised and fixed for 2 hours in 2% paraformaldehyde, then floated in 30% sucrose at 4^o^ overnight. Samples were then embedded in OCT compound (Tissue-Tek) and snap frozen. 5 µm sections were cut and stained with αVillin (clone 1D2C3, Santa Cruz), phalloidin (A12380; Invitrogen), and DRAQ5 (ThermoFisher). Confocal images were collected using a Nikon W1 spinning disk confocal microscope with a FusionBT SCMOS camera using a 100 × 1.45 N.A. objective lens.

### qRT-PCR analysis

Freshly isolated murine small intestinal crypts were collected and homogenized in TRIzol reagent (Thermo Fisher) using a 21 g needle. RNA was isolated using the Rneasy Mini Kit (Qiagen) with on-column DNAse digestion. cDNA was synthesized using the qScript cDNA synthesis kit (Quantabio). qPCR reactions were run using PerfeCTa SYBR Green SuperMix ROX (Quantabio) and primers designated in Supplementary Table S2. For analysis of human *MTGR1*, probes for *MTGR1* (Hs00602520_m1, Thermo Fisher) and *Gapdh* (Mm99999915_g1, Thermo Fisher) were used in conjunction with TaqMan Universal Master Mix II (Thermo Fisher). All samples were run in triplicate and target gene expression was analyzed using the delta–delta Ct method normalized to *Gapdh*.

### RNAscope

High-resolution RNA in situ hybridization was performed using the RNAscope® Multiplex Fluorescent V2 assay or RNAscope® 2.5HD Assay – Brown, according to the manufacturer’s instructions (ACDBio). Antigen retrieval was performed under standard pretreatment conditions as specified by the manufacturer. Probes were directed against mouse *Cbfa2t2* (#434601), *Lgr5* (#312171), or *Clu* (#427891). Fluorescent assay samples were mounted with ProLong Gold antifade reagent with DAPI and imaged as described above and images quantified by a blinded observer.

### Bulk RNA-sequencing

For RNA-sequencing, small intestine crypts were isolated from 3 WT and 3 *Mtgr1*^*−/−*^ mice. Following crypt isolation, a portion of the samples were collected and homogenized in TRIzol reagent while the remaining crypts were plated for enteroid culture. After 24 h, half of the plated enteroids were collected for RNA extraction, while the remaining enteroids were cultured for an additional 48 hours and harvested at 72 h post-plating. Additional samples were collected from passaged WT and *Mtgr1*^*−/−*^ enteroids, and RNA for all was isolated as described above. For RNA-sequencing studies, mRNA enrichment and cDNA library preparation were performed by the Vanderbilt Technologies for Advanced Genomics (VANTAGE) facility utilizing the Illumina Tru-seq stranded mRNA sample prep kit. Sequencing was performed at Single-Read 50 HT bp on the Illumina HiSeq 2500. Raw reads in FASTQ format were trimmed with fastp (v0.20.0) with default parameters [25]. Quantification was performed using Salmon (v1.3.0) [26] against a decoy transcriptome generated from Mus musculus GENCODE (v21) [27]. Further analysis was performed in R (v3.6.3) as described previously [28]. Briefly, quantification files were imported with tximeta (v1.4.5) [29]. Genes with counts ≤ 1 across all samples were omitted. Differential expression analysis (DEA) was performed on raw transcript counts using DESeq2 (v1.26.0) [30] and annotated with AnnotationDbi (v1.46.1) [31].

### Single cell RNA-sequencing data collection

Mouse tissues were used to generate single-cell RNA-seq data, following a methodology similar to previous studies ([32, 33] and Supplementary Methods). Single cell suspensions were generated via crypt isolation and digestion to single cells. The resulting cell suspensions underwent filtration, washing, and quality inspection before being encapsulated for microfluidic capture. The scRNA-seq procedure was carried out according to a modified protocol [34, 35]. Single-cell libraries were prepared for sequencing as detailed in previous documentation [36, 37]. These libraries, each containing an estimated 2000-3000 cell transcriptomes, were then sequenced on the Novaseq6000 platform, generating approximately 125 million reads per library.

### Single cell RNA-sequencing data analysis

Data quality was evaluated using ambiQuant [35]; data were then filtered using dropkick [38] and further processed according to an established pipeline ([39] and Supplementary Methods). Briefly, raw scRNA-seq counts were normalized by median library size, log-like transformed with arcsinh, and z-score standardized per gene followed by dimensional reduction and UMAP visualization using Python packages scanpy [40], pandas [41] and numpy [42]. To compare individual gene and gene signature expression between the wild-type and *Mtgr1* null groups, the two treatment groups underwent random sub-sampling to keep the same number of total cells in each group. Cell types of interest were extracted, and arcsinh-transformed counts were used for down-stream processes and GSEA using gene sets in the WikiPathways_2019_Mouse library. Relevant code is available at https://github.com/Ken-Lau-Lab/MTGR1_SI_stem_cell_analysis.git.

### Electron microscopy

Specimens were processed for transmission electron microscopy (TEM) and imaged in the Vanderbilt Cell Imaging Shared Resource: Research Electron Microscopy facility according to their established methods (Supplementary Methods). Following processing and epoxy embedding, 70–80 nm ultra-thin sections were cut, collected on 300-mesh copper grids, and post-stained with 2% uranyl acetate followed by Reynold’s lead citrate. Samples were subsequently imaged on the Philips/FEI Tecnai T12 electron microscope.

### Lgr5-EGFP+ cell isolation and sorting

Single cell suspensions were generated from duodenal crypt cells, and flow cytometry for *Lgr5-EGFP* was performed as previously described ([43] and Supplementary Methods). Notably, 10 μM ROCK inhibitor (Y-27632) (#12-541-0, Fisher Scientific) was added to all buffers and solutions to maximize cell viability. Cell suspensions were stained with 1:100 APC Annexin V (#640919 BioLegend) and 1:4000 propidium iodide (PI) (#281487-000, Invitrogen) prior to FACS analysis in collaboration with the Vanderbilt Flow Cytometry Shared Resource.

### Statistics

Unless noted, statistical analysis was performed in Graphpad Prism 8 Software using Student’s t-test (unpaired, two-tailed) or Mann-Whitney Wilcoxon test for single comparisons pending results of data normality. Kruskal-Wallis test with Dunn’s multiple comparison test was used for multiple samples, or two-way ANOVA (repeated measures) and Sidak’s multiple comparison post-test for time course analyses. Samples were excluded if determined to be statistical outliers based on “robust regression and outlier removal” (ROUT) analysis. Center values represent the median for violin plots, but for all studies center values represent experimental mean. Error is represented by standard error of the mean and *P* < 0.05 is considered significant. Sample sizes are delineated in relevant figure legends and determined by prior experience using these models and generally accepted number of replicates for equivalent types of studies.

## Results

### MTGR1 is widely expressed in the intestine

**Fig. 1:**
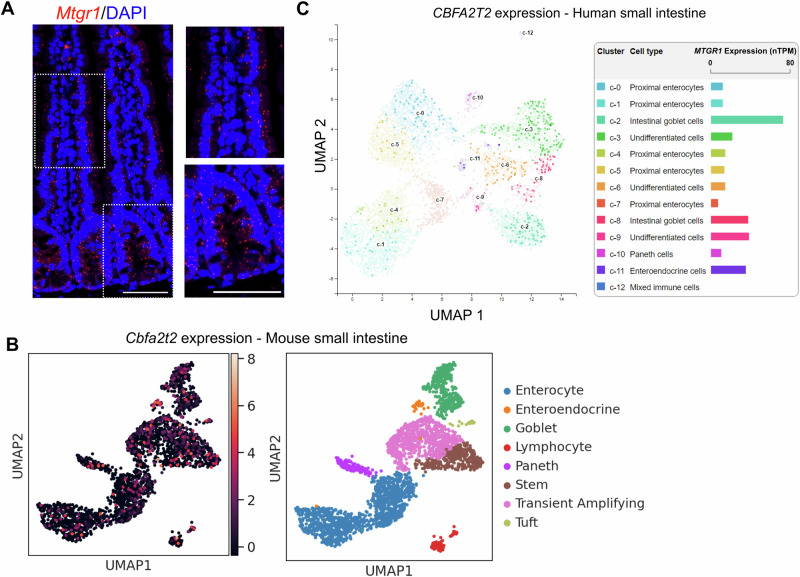
MTGR1 is widely expressed in the small intestine. **A** *Mtgr1* mRNA assessed by RNAScope in the WT small intestine. Results representative of 3 independent experiments. Dotted lines indicate inset areas. Scale bar = 100 µm. **B** Uniform manifold approximation and projections (UMAPs) showing *Mtgr1* (*Cbfa2t2*) expression in the murine ileum by scRNA-sequencing (left) and associated cell clusters (right). n = 2 mice. **C** Human *MTGR1 (CBFA2T2)* expression was queried from the Human Protein Atlas scRNA-sequencing data. *MTGR1* expression is visualized by UMAP (left) and bar graphs (right) in various intestinal cell types.

Work by our group and others has identified key roles for MTG family members in promoting secretory differentiation. In line with this role, MTG16 is enriched in specific secretory cell populations as well as +4/+5 intestinal cells, often thought to be both reserve stem cells and secretory lineage progenitors [15, 21]. However, the expression pattern of MTGR1 in intestinal cell populations is undefined. To define MTGR1 expression patterns, we first utilized in situ staining to spatially visualize *Mtgr1* transcripts in the murine small intestine (Fig. 1A). Here, *Mtgr1* expression was dispersed throughout the intestinal crypt-villus axis and did not appear to be specifically localized to distinct cell populations [15]. We next investigated *Mtgr1* expression in specific cell types via single-cell RNA-sequencing (scRNA-seq) of the murine ileum and jejunum [32, 44]. These results confirmed widespread *Mtgr1* expression in various intestinal cells that was not restricted to specific cellular lineages (Fig. 1B and Supplementary Fig. 1). Similar results were observed in the human small intestine through query of publicly available scRNA-seq data from the Human Protein Atlas (GSE125970), with *MTGR1* expression again observed in multiple differentiated and undifferentiated cell types (Fig. 1C) [45, 46]. Thus, *Mtgr1* is widely expressed throughout the intestinal epithelium and not restricted to secretory lineage cells and progenitors.

### MTGR1 loss expands ISC populations in vivo

**Fig. 2:**
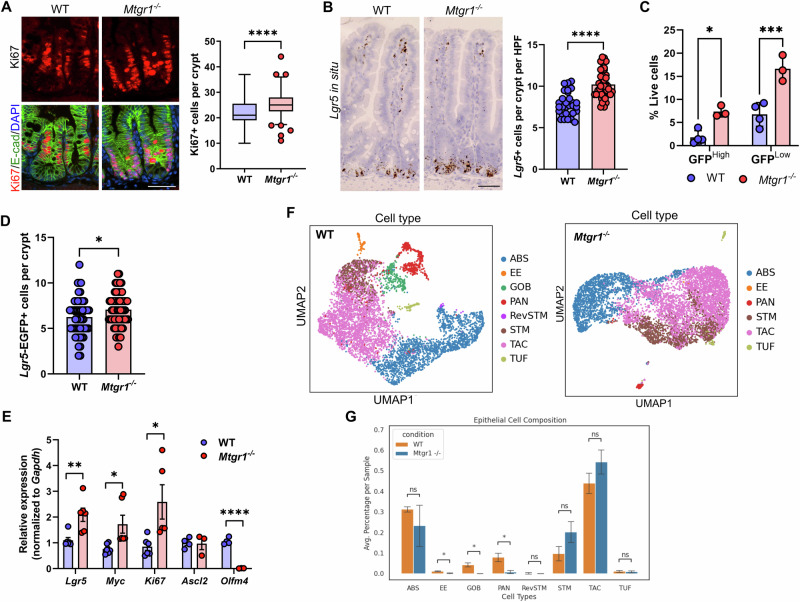
MTGR1 loss increases ISC number and deregulates intestinal stem cell programs. **A** Immunofluorescent staining for Ki67 (red), E-cadherin (green), and nuclei (DAPI, blue) in the small intestine of 8–12-week-old WT and *Mtgr1*^*−/−*^ mice. n = 4 mice per genotype, >20 high-powered fields (HPFs) per mouse. **B** *Lgr5* mRNA expression was visualized in the WT and *Mtgr1*^*−/−*^ small intestine by RNAscope. n = 4 WT and 3 *Mtgr1*^*−/−*^ mice, 12 HPFs per mouse. **C** *Mtgr1*^*−/−*^ mice were intercrossed with the *Lgr5-cre-EGFP* reporter strain and isolated crypt cells were stratified by *Lgr5*-EGFP expression through FACS. n = 4 WT and 3 *Mtgr1*^*−/−*^ mice. **D** *Lgr5*-EGFP assessed by immunofluorescence. Quantification shows the number of GFP positive cells in each reporter positive crypt, per mouse. n = 9 mice per genotype. **E** q-RT-PCR of stem cell markers *Lgr5*, *Myc*, *Ki67, Ascl2*, and *Olfm4* in intestinal crypt isolates from *Mtgr1*^*−/−*^ and WT mice (n = 3–6 mice per genotype). Results were normalized to *Gapdh* and represented as fold change over WT expression. **F** UMAPs depicting cell types as determined from scRNA-sequencing results from WT and *Mtgr1*^*−/−*^ intestinal cells and (**G**) numerical representation. n = 3 WT and 2 *Mtgr1*^*−/−*^ duodenal samples. ABS absorptive, EE enteroendocrine, GOB goblet, PAN Paneth, RevSTM revival stem, STM stem, TAC transit amplifying cell, TUF tuft. *P < 0.05, **P < 0.01, ***P < 0.001, ****P < 0.0001, Mann–Whitney test (**A**, **D**, select **E**), Student’s t test (**B**, select **E**), or two-way ANOVA with Sidak’s multiple comparison test (**C**), Scale bars = 100 µm.

Interestingly, higher levels of intestinal epithelial proliferation have been reported in mice globally lacking MTGR1 (*Mtgr1*^*−/−*^), suggesting MTGR1 expression may regulate undifferentiated ISC populations in addition to secretory lineages[14, 18, 47]. First, we again confirmed the expansion of proliferative cells in the crypts of *Mtgr1*^*−/−*^ mice, here via quantifying Ki67 expression (Fig. 2A). We next hypothesized that this increase in proliferation may be associated with higher numbers of LGR5+ ISCs, due to their role in maintaining proliferation in the intestine at baseline [14, 18, 47]. *Lgr5*-expressing cells in the small intestine were identified by in situ hybridization. Here, we determined that *Mtgr1*^*−/−*^ mice indeed had higher numbers of *Lgr5*+ cells per crypt as compared to WT mice (Fig. 2B). Intercross of *Mtgr1*^*−/−*^ mice with the *Lgr5-Cre-EGFP* reporter line, which expresses EGFP from the *Lgr5* locus, also showed increased numbers of both high and low-expressing *Lgr5*-EGFP+ cells in *Mtgr1*^*−/−*^ versus WT *Lgr5-Cre-EGFP* mice (Fig. 2C). Due to the mosaic expression of the *Lgr5* reporter, increases in *Lgr5*+ cells were confirmed by restricting quantification to reporter positive crypts by immunofluorescent staining against GFP (Fig. 2D). Finally, higher levels of *Lgr5*, *Ki67*, and *Myc* transcripts were also observed in *Mtgr1*^*−/−*^ crypt isolates by q-RT-PCR (Fig. 2E).

While *Lgr5* is often regarded as the canonical identifier of CBCs and a robust ISC marker, we next assayed for expression of other ISC-associated genes. For example, *Ascl2* and *Olfm4* are highly expressed in CBCs along with *Lgr5* [48, 49]. Interestingly, although *Lgr5* transcript and *Lgr5*-EGFP+ cells were consistently increased with MTGR1 loss, neither *Ascl2* nor *Olfm4* mirrored these changes. Here, we observed that the *Ascl2* transcript remained unchanged in *Mtgr1*^*−/−*^ crypts, while *Olfm4* was nearly undetectable in *Mtgr1*^*−/−*^ crypts (Fig. 2E), suggesting broad deregulation of ISC programs in context of MTGR1 loss. Next, to more comprehensively map changes in intestinal cell populations, we performed single cell RNA sequencing (scRNA-seq) on WT and *Mtgr1*^*−/−*^ duodenal cells. Following UMAP dimension reduction and cell lineage stratification according to our established methods [32, 33, 44], MTGR1 loss was observed to have wide-ranging effects on intestinal cell differentiation (Fig. 2F, G and Supplementary Fig. 2). As observed previously, *Mtgr1*^*−/−*^ mice broadly lack secretory Paneth, enteroendocrine, and goblet cells. However, we observed a trend towards increased ISC and TA populations. Together with results from the *Lgr5-EGFP* reporter mouse, these studies indicate that MTGR1 loss expands total ISC cell numbers in the small intestine.

### MTGR1 is required for enteroid viability

**Fig. 3:**
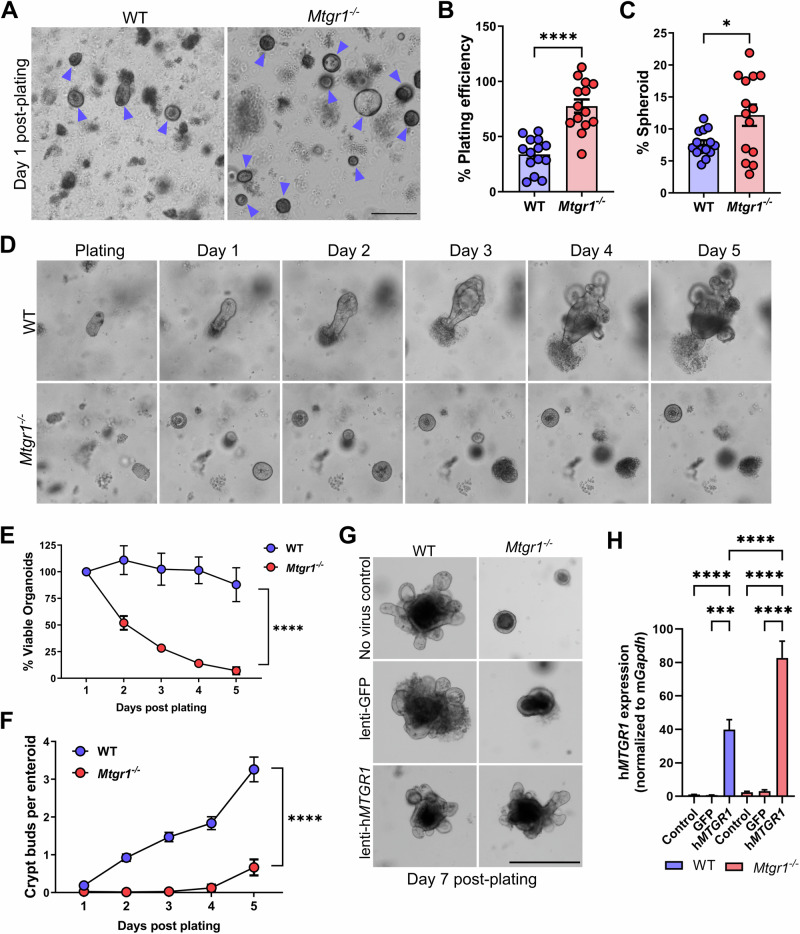
MTGR1 loss increases initial plating efficiency but is not compatible with enteroid survival. **A** Crypts were isolated from WT and *Mtgr1*^*−/−*^ mice and plated as intestinal enteroids. Representative images of enteroids at day 1 post-plating with enteroids marked by blue arrows. Scale bar = 200 µm (left), (**A**–**E**) representative of 4 independent experiments. **B** Quantification of overall plating efficiency (enteroids established divided by crypts plated) and (**C**) percentage of enteroids with cystic morphology calculated per well at day 1 post-plating. n = 14 wells per genotype. **D** 5-day timelapse imaging of WT and *Mtgr1*^*−/−*^ enteroids. **E** Average enteroid viability post-plating, shown as the percent viable enteroids remaining from day 1. n = averaged 4 independent experiments per genotype. **F** Quantification of crypt budding post-plating. n = 75, 67, 74, 68, and 26 WT enteroids and n = 136, 121, 70, 16, and 6 *Mtgr1*^*−/−*^ enteroids. **G** WT and *Mtgr1*^*−/−*^ crypts were transduced with lentiviral GFP or human *MTGR1* and plated to allow enteroid formation. Representative images at day 7 post-infection/plating. Scale bars = 500 µm. **H** Transduced enteroids were collected at day 7 post-infection for mRNA analysis of human *MTGR1*. Results were normalized to *Gapdh* and shown as fold change over WT non-transduced controls. n = 2–3 independent experiments for addback studies. *P < 0.05, **P < 0.01, ***P < 0.001, ****P < 0.0001, Student’s t test (**B**, **C**), two-way ANOVA (**E**, **F**), or one-way ANOVA (**H**).

As loss of MTGR1 increased *Lgr5* + ISCs, and MTGs have been noted to dampen Wnt pathway activity through interaction with TCF4 [18], we next hypothesized that MTGR1 loss would increase ISC number and function. Here, we utilized the small intestinal organoid or “enteroid” system. Since enteroids rely on ISCs for their establishment and growth, enteroid formation efficiency can be used to assess general stem cell function and fitness [3]. Here, enteroids were established from duodenal crypts harvested from WT and *Mtgr1*^*−/−*^ mice, and enteroid formation efficiency was assessed after 24 h in culture (Fig. 3A, B). By dividing the number of enteroids formed by the number of crypts plated, we noted an approximately twofold enhancement of enteroid formation in the setting of MTGR1 loss. We also observed higher percentages of *Mtgr1*^*−/−*^ enteroids with a cystic, spheroid morphology (Fig. 3C), a phenotype associated with increased Wnt tone [19], compared to WT enteroids.

Despite the initial augmentation of enteroid formation in *Mtgr1*^*−/−*^ cultures, we observed striking viability defects in *Mtgr1*^*−/−*^ enteroids within 48 h of initial plating. Daily imaging (Fig. 3D) and viable enteroid counts (Fig. 3E) revealed that *Mtgr1*^*−/−*^ cultures failed almost completely by day 5 post-plating. While WT enteroids formed crypt buds by day 3, *Mtgr1*^*−/−*^ enteroids rarely developed crypt buds, even in the structures that survived until day 5 (Fig. 3F). These findings were also confirmed by live cell imaging, which showed no morphological changes in *Mtgr1*^*−/−*^ enteroids throughout the 5-day period, until enteroid death (Supplementary videos SV1 and SV2). Importantly, restoration of *MTGR1* expression via lentiviral transduction rescued *Mtgr1*^*−/−*^ enteroids and restored branching morphology, confirming the MTGR1 dependency of this phenotype (Fig. 3G, H). Thus, MTGR1 appears to be required for ex vivo enteroid survival and expansion.

### Inhibition of cell death pathways does not rescue Mtgr1^−/−^ viability

**Fig. 4:**
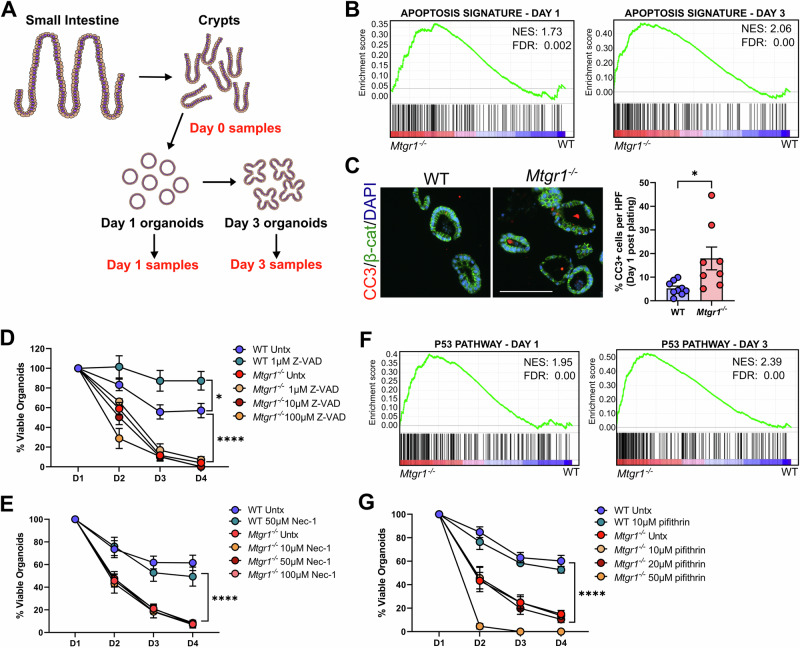
MTGR1-dependent enteroid loss cannot be rescued by inhibition of cell death pathways. **A** Schematic of RNA-sequencing experiment of crypts and enteroids. n = 3 mice per genotype per timepoint. **B** GSEA of “Hallmark” collection apoptosis-related genes in *Mtgr1*^*−/−*^ enteroids at day 1 (left) and day 3 (right) post-plating. NES = normalized enrichment score. **C** Enteroids were fixed and embedded at day 1 post-plating, and apoptotic cells were marked by immunofluorescent staining against cleaved caspase-3 (CC3, red). β-catenin (green) and DAPI (blue) were used for co-staining. Quantification shown as percent CC3-positive cells per high powered field (HPF). Scale bar = 200 µm. n = 9 WT and 8 *Mtgr1*^*−/−*^ HPFs. **D** Enteroids were plated and overlaid with media containing indicated concentrations of the caspase inhibitor, Z-VAD-FMK, or (**E**) the necrosis inhibitor, necrostatin. Enteroids were counted daily and normalized to day 1 numbers. n = 6 wells per condition. **F** GSEA of “Hallmark” collection p53 pathway-related genes in *Mtgr1*^*−/−*^ enteroids at day 1 (left) and day 3 (right) post-plating. **G** Enteroids were plated and overlaid with media containing the p53 inhibitor, pifithrin, as indicated. n = 6 WT and 12 untreated, 9 (10 µm), 10 (20 µm), and 3 (50 µm) *Mtgr1*^*−/−*^ enteroid wells. *P < 0.05, ****P < 0.0001, Student’s t test (**C**) or two-way ANOVA (**D**, **E**, **G**), significance indicated by FDR q value (**B**, **F**).

We next aimed to determine the mechanism driving the viability loss in *Mtgr1*^*−/−*^ enteroids. As MTGR1 is a transcriptional co-repressor, we utilized a bulk RNA-sequencing approach to broadly investigate MTGR1-dependent changes in gene expression. Briefly, crypts were isolated from age-matched WT and *Mtgr1*^*−/−*^ mice, and mRNA was collected at the time of crypt isolation (day 0) or at day 1 and day 3 post-plating to yield matched crypt, day 1 enteroid, and day 3 enteroid mRNA (Fig. 4A). After RNA-sequencing, differential expression profiles were generated and analyzed using gene set enrichment analysis (GSEA) [50, 51].

Due to the rapid loss of established cultures and minor increases in apoptosis previously reported from *Mtgr1* null crypts [14], we hypothesized that MTGR1 loss may aberrantly activate programmed cell death pathways to decrease enteroid viability. Indeed, GSEA analysis from the Hallmark gene set collection identified a significant enrichment in apoptosis-associated genes in *Mtgr1*^*−/−*^ enteroids at both day 1 and day 3 post-plating (Fig. 4B). *Mtgr1*^*−/−*^ enteroids collected at day 1 post-plating also displayed a modest increase in the percentage of apoptotic cells as measured by fluorescent immunohistochemistry (IHC) against cleaved caspase-3 (Fig. 4C). However, inhibiting apoptosis using the cell-permeable pan-caspase inhibitor, Z-VAD-FMK, failed to improve survival of *Mtgr1*^*−/−*^ enteroids (Fig. 4D), even at concentrations which improved viability in WT cultures [52]. Likewise, inhibition of necroptosis, which is dysregulated in intestinal inflammatory diseases [53–55], had no effect on *Mtgr1*^*−/−*^ enteroid viability (Fig. 4E). Finally, we assessed the impact of p53 inhibition, as p53-related gene sets were also positively enriched in *Mtgr1*^*−/−*^ samples by GSEA (Fig. 4F). As with Z-VAD-FMK, inhibition of p53-dependent apoptosis with pifithrin-α had no effect on *Mtgr1*^*−/−*^ enteroid survival (Fig. 4G) [56]. Thus, inhibition of these cell death mechanisms is insufficient to rescue *Mtgr1*^*−/−*^ enteroid viability, suggesting that altered apoptotic responses are unlikely to be the underlying cause of enteroid failure.

### Proliferation and ISC-associated genes are lost in MTGR1-deficient enteroids

**Fig. 5:**
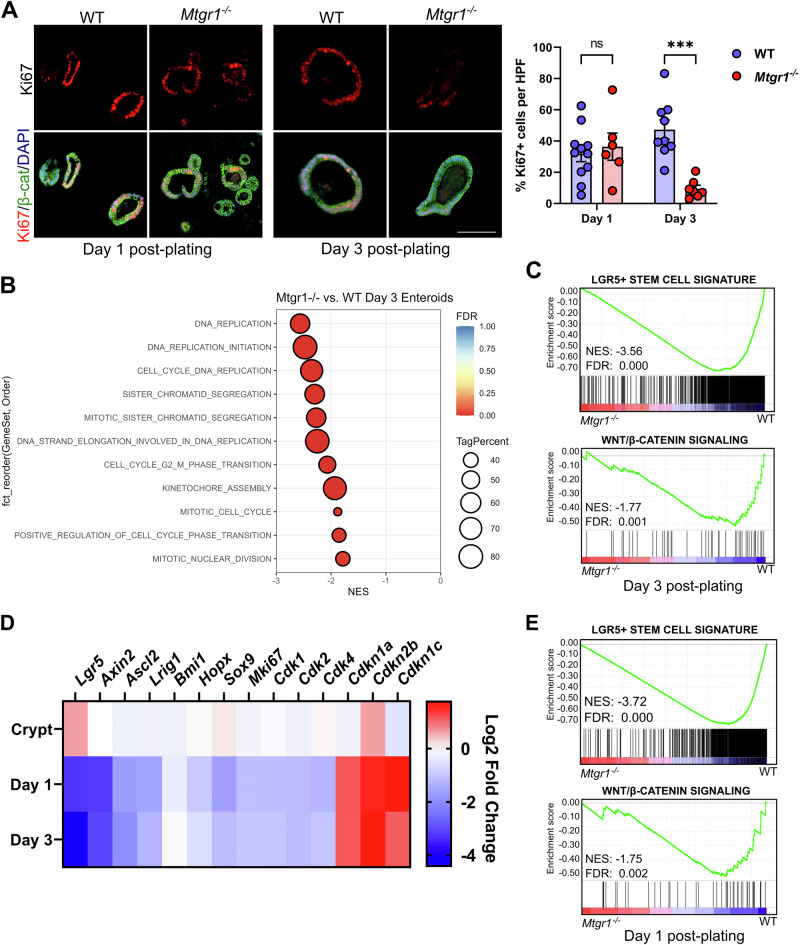
MTGR1 loss decreases proliferation and stem cell-associated gene expression during enteroid maturation. **A** WT and *Mtgr1*^*−/−*^ enteroids were fixed at day 1 and day 3 post-plating and proliferative cells were marked via immunohistochemistry for Ki67 (red). β-catenin (green) and DAPI (blue) were used for co-staining. Quantification shown as percent Ki67-positive cells per high powered field (HPF). Scale bar = 200 µm. n = 11 or 9 WT and 6 or 7 *Mtgr1*^*−/−*^ HFPs per timepoint. **B** Gene set enrichment analysis (GSEA) of day 3 RNA-sequencing results using cell cycle-related gene sets queried from the Gene Ontology collection. NES = normalized enrichment score. Tag % = the percentage of gene hits before (for positive ES) or after (for negative ES) the peak in the running ES, indicating the percentage of genes contributing to the ES. **C** GSEA of day 3 RNA-sequencing results with intestinal stem cell- (top) and Wnt-associated (bottom) gene sets. **D** Heatmap of RNA-sequencing results of stem cell, cyclin dependent kinases, and cyclin-dependent kinase inhibitors from crypt, day 1, and day 3 results. Represented as the Log2 fold change of *Mtgr1*^*−/−*^ results as compared to WT at that timepoint. **E** GSEA of day 1 RNA-sequencing results with intestinal stem cell- (top) and Wnt-associated (bottom) gene sets. ns = nonsignificant, ***P < 0.001, Student’s t test (**A**), significance indicated by FDR q value (**B**, **C**, **E**).

Due to constant cell clearance, actively cycling stem cells and high levels of proliferation are necessary to maintain intestinal cell populations [57]. Thus, rather than aberrant apoptosis, we next hypothesized that the viability defect in *Mtgr1*^*−/−*^ enteroids may instead be due to reduced proliferation and/or depletion of ISCs. To determine cell proliferation, sections from enteroids embedded at day 1 and day 3 post-plating were assessed by Ki67 IHC (Fig. 5A). Although we observed similar numbers of proliferating cells in day 1 enteroids, by day 3, the enteroid cultures established from *Mtgr1*^*−/−*^ mice displayed a drastic, nearly 80% reduction in Ki67+ cells. Cell cycle- and proliferation-associated genes were also highly downregulated in *Mtgr1*^*−/−*^ enteroids by day 3 (Fig. 5B), as well as ISC-associated genes and signaling pathways (Fig. 5C), as determined by GSEA. Interestingly, while numbers of Ki67+ cells were similar between WT and *Mtgr1*^*−/−*^ enteroids at day 1 post-plating, proliferation-, ISC-, and Wnt-associated genes were still significantly downregulated at this early timepoint (Fig. 5D, E, and Supplementary Table S3). *Mtgr1*^*−/−*^ enteroids, at either day 1 or day 3 post-plating, also demonstrated significant upregulation of the cell cycle inhibitors *Cdkn1a*, *Cdkn1c*, and *Cdkn2b*. These results indicate that viability defects in *Mtgr1*^*−/−*^ enteroids may arise from proliferation defects and the inability to maintain cycling ISC populations ex vivo.

### Terminal differentiation likely drives MTGR1-dependent enteroid loss

**Fig. 6:**
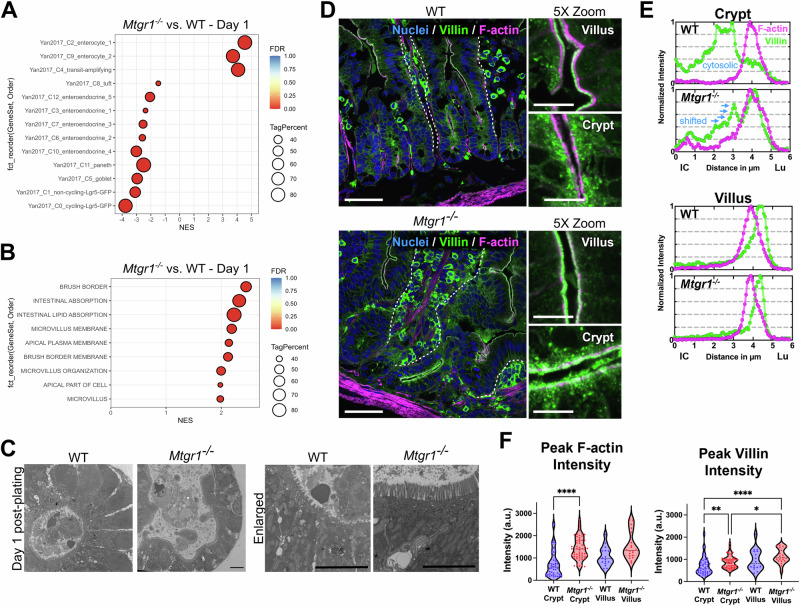
MTGR1 loss promotes absorptive differentiation. **A** Gene set enrichment analysis (GSEA) of *Mtgr1*^*−/−*^ day 1 enteroid RNA-sequencing results using gene sets representing intestinal epithelial cell types. NES = normalized enrichment score. Tag % = the percentage of gene hits before (for positive ES) or after (for negative ES) the peak in the running ES, indicating the percentage of genes contributing to the ES. Significance indicated by FDR q value. **B** GSEA of day 1 *Mtgr1*^*−/−*^ RNA-sequencing results using gene sets representing microvilli and brush border biology queried from the Gene Ontology collection at day 1 post plating. **C** Representative electron microscopy images from WT and *Mtgr1*^*−/−*^ day 1 enteroid samples, n = 2 per genotype. Scale bar = 5 µm. **D** Representative confocal images from WT and *Mtgr1*^*−/−*^ mouse duodenal tissue stained with DRAQ5 (blue, nuclei), Villin (green), and phalloidin (F-actin, magenta). Dotted lines designate basal epithelial border on villi. Enlarged images (5x zoom) highlight crypt and villus surfaces. Main panel scale bars 50 µm, 5× zoom scale bars 10 µm. **E** Representative normalized line scans showing F-actin (magenta) and Villin (green) intensities. Line scans are oriented from intracellular (IC, distance = 0) to the intestinal lumen (Lu). **F** Quantification of peak F-actin intensity (left) and peak Villin intensity (right) on crypt and villus epithelial surfaces. Data represents peak background subtracted intensity from a minimum of 15 line-scans obtained from at least 5 separate crypts/villi and tissue from 3 separate mice. *P < 0.05, **P < 0.01, ****P < 0.0001. Significance indicated by FDR q value (**A**, **B**) or Kruskal–Wallis test (**F**).

After expansion in the TA zone and exit from the intestinal crypt, most ISC-derived cells rapidly undergo differentiation into non-proliferative cell lineages [58]. As cell proliferation and ISC-associated signaling are reduced in *Mtgr1*^*−/−*^ enteroids, we next hypothesized that the loss of enteroid viability may be due to terminal differentiation into non-proliferative cells and the inability to sustain further enteroid growth. First, to more clearly assess the overall differentiation status of WT and *Mtgr1*^*−/−*^ enteroids, we overlaid our bulk RNA-sequencing results with established gene sets associated with specific intestinal cell types [8]. Here, these data indicate significant enrichment of genes associated with enterocyte populations in day 1 enteroids, and also reflect expected decreases in secretory and ISC-associated genes (Fig. 6A). Likewise, GSEA analysis determined significant enrichment of genes associated with features of absorptive enterocytes, such as the brush border, microvilli, and intestinal absorption at both day 1 (Fig. 6B) and day 3 post-plating (Supplementary Fig. 3). Next, comparison of the cellular structure of day 1 enteroids was investigated using transmission electron microscopy (TEM), which further illustrates an expansion of the apical cell surface as well as more pronounced and mature microvilli in *Mtgr1*^*−/−*^ enteroids (Fig. 6C). Together with the previous results which show lowered proliferation and Wnt pathway activity, these data indicate rapid differentiation of stem cells into enterocytes in *Mtgr1*^*−/−*^ enteroids. Ultimately, this would preclude further ex vivo culture due to lack of ISCs.

We aimed to confirm whether loss of MTGR1 also promotes absorptive differentiation in intestinal crypts, in vivo. As observed in *Mtgr1*^*−/−*^ enteroids, transcriptomic analysis again determined enrichment of enterocyte related gene sets in *Mtgr1*^*−/−*^ duodenal crypts as compared to WT (Supplementary Fig. 4). To visualize brush border differentiation at the crypt-villus transition, we conducted confocal imaging of Villin and F-actin, core components of the intestinal brush border (Fig. 6D) [59]. Villin typically has an apical cytosolic distribution within crypts, then incorporates into forming microvilli while concentrating in the mature brush border during absorptive differentiation [60–62]. Representative line-scans in the crypts of *Mtgr1*^*−/−*^ mice highlight that Villin shifted toward the apical surface with the peak intensity overlapping with the F-actin signal. This difference was not observed in the villi, where F-actin and Villin localization are normal, with peak Villin intensity distal to the peak F-Actin intensity, and lower cytosolic Villin (Fig. 6E). Likewise, greater peak staining intensity was observed for both Villin and F-actin in *Mtgr1*^*−/−*^ crypts as compared to WT (Fig. 6F). Taken together, these results indicate that loss of MTGR1 strongly promotes differentiation into absorptive enterocytes and suggests crypt cells from *Mtgr1*^*−/−*^ mice are more differentiated than WT crypt cells.

### Secretory cells promote the survival of Mtgr1^−/−^ enteroids, but do not fully rescue

We next hypothesized that stabilizing stem cell function and inhibiting this rapid absorptive differentiation would rescue MTGR1-dependent enteroid death. MTGR1 is necessary for the differentiation of multiple secretory lineages (Fig. 2G) [14, 17], and Paneth cells are crucial regulators of ISCs and provide Wnt ligands that maintain ISC stemness and multipotency [63]. Furthermore, crypts from mice lacking Paneth cells cannot form enteroid cultures without Wnt supplementation [63–66]. Therefore, decreased Paneth cells may contribute to the loss of *Mtgr1*^*−/−*^ enteroids via decreased ISC support.

**Fig. 7:**
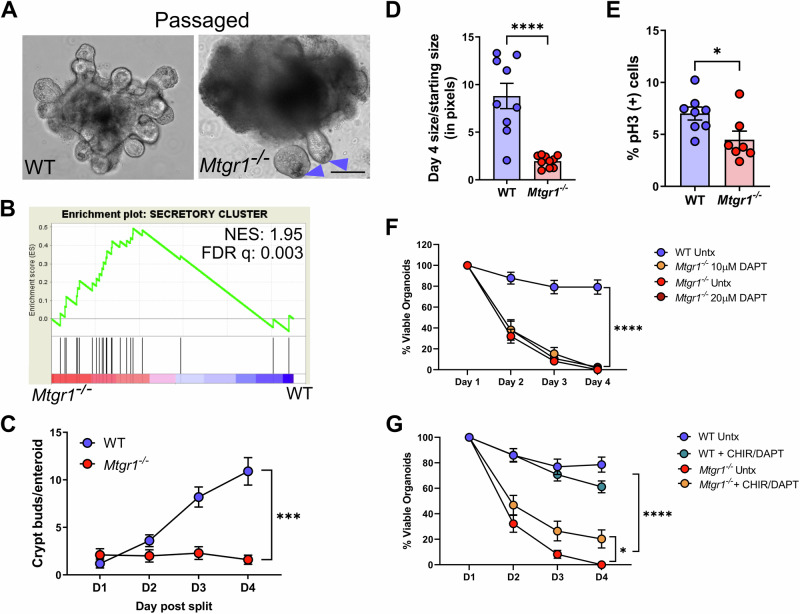
Secretory differentiation promotes, but does not rescue, survival of *Mtgr1* null enteroids. **A** Representative images showing passaged WT enteroids and *Mtgr1*^*−/−*^ enteroids. Passaged WT and *Mtgr1*^*−/−*^ enteroids both had discernable Paneth cells in the crypt base (arrows). Scale bar = 200 µm. **B** Gene set enrichment analysis (GSEA) of passaged *Mtgr1*^*−/−*^ enteroids against secretory cell associated genes. NES normalized enrichment score. Significance indicated by FDR q value. **C** Quantification of crypt buds per passaged enteroid post-split, n = 10 enteroids per genotype. **D** Passaged enteroids were imaged at day 1 and day 4 post-passage and enteroid area measured via ImageJ. Change in size was calculated by dividing day 4 measurements by those taken at day 1. n = 10 enteroids per genotype. **E** Passaged *Mtgr1*^*−/−*^ enteroids were fixed and stained with phospho-histone H3 (pH3) to mark proliferative cells. Quantification shown as percent pH3-positive cells per high powered field (HPF). n = 8 WT and 7 *Mtgr1*^*−/−*^ HPFs. **F** Enteroids were plated and overlaid with media containing indicated concentrations of the gamma secretase inhibitor, DAPT. Enteroids were counted daily and normalized to day 1 numbers. n = 7 WT and 8 *Mtgr1*^*−/−*^ wells per condition. **G** WT and *Mtgr1*^*−/−*^ enteroids were plated and supplemented with 3 µM of CHIR-99021 (CHIR) and 10 µM DAPT. Enteroids were counted daily and normalized to day 1 numbers. n = 8 wells per condition. Enteroid numbers were assessed daily and normalized to day 1 results. *P < 0.05, ***P < 0.001, ****P < 0.0001, significance indicated by FDR q value (**B**), two-way ANOVA (**C**, **F**, **G**), and Student’s t test (**D**, **E**).

While the majority of enteroids from *Mtgr1*^*−/−*^ mice died by day 5, a small number of surviving enteroids occasionally could be maintained and passaged. Interestingly, in these cultures, Paneth cells could often be distinguished in the crypt base (Fig. 7A), and secretory cell-associated genes were highly expressed, proportionally, via transcriptomic analysis (Fig. 7B). However, while Paneth cells were associated with these surviving *Mtgr1*^*−/−*^ enteroids, passaged *Mtgr1*^*−/−*^ enteroids still displayed striking alterations in morphology: an inability to form enteroid buds (Fig. 7C), little expansion in size over time (Fig. 7D), low proliferation as measured by immunofluorescent IHC against the proliferative marker phospho-histone H3 (pH3, Fig. 7E), and lower levels of ISC-associated signaling (Fig. 7F). Thus, while these Paneth-containing enteroids can survive, they still display growth, morphology, and ISC defects.

We next employed various combinations of small molecules in an attempt to more widely promote secretory cell differentiation and survival of *Mtgr1*^*−/−*^ ISCs. First, we utilized DAPT, a γ-secretase inhibitor that increased secretory cell numbers ex vivo at the expense of absorptive lineages [17]. However, γ-secretase inhibitor treatment failed to rescue *Mtgr1*^*−/−*^ enteroid growth ex vivo (Fig. 7G), even when treatment was begun in vivo prior to and continuing through enteroid establishment (data not shown). Next, we combined DAPT with the Wnt pathway agonist, CHIR 99021, as this combination should greatly promote Paneth cell differentiation [67]. These studies revealed only a modest increase in enteroid survival (Fig. 7H). Paneth cell-deficient enteroids can be rescued by co-culture with intestinal stroma or WT enteroids; however, these co-culture strategies likewise failed to rescue *Mtgr1*^*−/−*^ enteroid survival (Supplementary Fig. 5) [64–66]. Taken together, these results suggest that the function of secretory lineage cells, alone, cannot rescue *Mtgr1* deficient enteroid survival.

### Defective Mtgr1^−/−^ ISCs require hyper Wnt stimulation

**Fig. 8:**
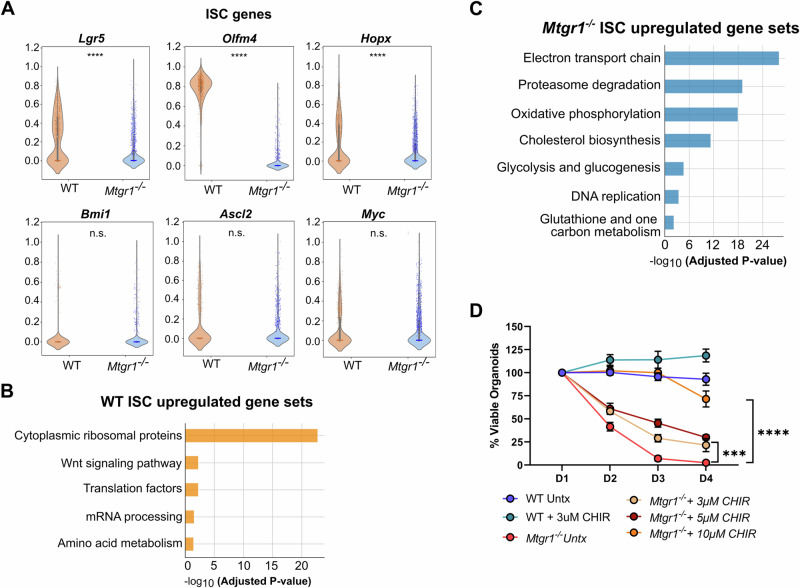
Deficient *Mtgr1*^*−/−*^ ISCs can be rescued by high Wnt stimulation. **A** ISC cells identified by scRNA-seq were queried for expression of ISC associated genes. **B** ISC-specific transcriptomic signatures were assessed by GSEA using the WikiPathways_2019_Mouse collection. Subset of statistically significant gene sets/pathways shown here (all significant pathways shown in Supplementary Fig. 7) for WT and (**C**) *Mtgr1*^*−/−*^ ISCs. **D** WT and *Mtgr1*^*−/−*^ enteroids were plated with the indicated amounts of CHIR. Enteroid numbers were assessed daily and normalized to day 1 results. n = 7 WT and 10 *Mtgr1*^*−/−*^ control samples, 4 samples for all other genotypes/conditions. ***P < 0.001, ****P < 0.0001, Mann–Whitney Wilcoxon test (**A**), significance indicated by FDR q value (**B**, **C**), or two-way ANOVA (**D**).

As growth and differentiation defects could not be rescued by any mechanism tested thus far, we next hypothesized that MTGR1-dependent changes in ISCs may ultimately be responsible for the failure of *Mtgr1*^*−/−*^ enteroids to expand ex vivo, despite the increase in *Lgr5+* cells and initial greater enteroid establishment. Therefore, we specifically investigated transcriptomic changes in ISC cells as identified by scRNA-sequencing. Surprisingly, *Lgr5* was noted to be reduced on a per cell basis, as were *Hopx* and *Olfm4* (Fig. 8A and Supplementary Fig. 6). GSEA revealed enrichment of genes involved in Wnt signaling in WT crypts (Fig. 8B and Supplementary Fig. 7). *Mtgr1*^*−/−*^ ISCs, on the other hand, were highly enriched in transcripts associated with oxidative phosphorylation, a shift to which is associated with ISC differentiation and absorptive intestinal cells [68, 69] (Fig. 8C and Supplementary Fig. 7). Thus, it seems likely that *Mtgr1*^*−/−*^ ISCs are inherently functionally deficient and increased total numbers *Lgr5*+ cells are necessary to maintain intestinal homeostasis.

Finally, we investigated whether Wnt pathway activation alone was sufficient to maintain *Mtgr1*^*−/−*^ ISCs. CHIR 99021 treatment at a concentration sufficient to promote WT ISCs and compensate for Paneth cell loss (3 µM) only had a modest effect on *Mtgr1*^*−/−*^ enteroid survival (Fig. 8D) [70]. However, enteroid failure could finally be overcome by increasing the concentration of CHIR 99021 to 10 µM, which sustained long-term survival and growth of *Mtgr1*^*−/−*^ ISCs ex vivo (Fig. 8D). Altogether, these results indicate that while MTGR1 loss expands *Lgr5* + ISC populations, *Lgr5* transcripts are reduced on a per cell basis, and that MTGR1 is necessary for maintaining ISC function in the small intestine.

## Discussion

In this study, we establish that MTGR1 promotes absorptive differentiation and is required for the proper function of ISCs. While MTGR1 loss led to increased proliferation and *Lgr5+* stem cell numbers, our data indicate that individual *Mtgr1*^*−/−*^ ISCs, themselves, had reduced levels of ISC-associated gene pathways and were unable to sustain ex vivo culture. Indeed, survival of *Mtgr1*^*−/−*^ enteroids was only possible through inducing high Wnt pathway activity, indicating that strong stem cell-promoting signals are necessary to correct cell-intrinsic defects of *Mtgr1* deficiency.

MTGR1 belongs to the Myeloid Translocation Gene family, which have been widely implicated in stem cell maintenance and lineage commitment in the hematopoietic system and beyond [12, 15, 71]. In the homeostatic intestine, *Mtg16*^*−/−*^ and *Mtgr1*^*−/−*^ mice both display increased proliferation and altered secretory lineage allocation [14, 15, 17, 21, 47, 71]. However, while the MTG family members share significant sequence homology and generally similar phenotypes, our data highlights their diverse effects in the gut, particularly in specific cell types. For example, while loss of MTGR1 depleted nearly all intestinal secretory cells, loss of MTG16 appeared to regulate more subtle cell fate decisions between goblet cells and EECs in the colon [21]. Meanwhile, it has recently been reported that MTG16 and MTG8 are both enriched in +4/+5 cells, where they repress ISC-specific genes to control exit from the stem cell niche [15]. On the other hand, enrichment of MTGR1 was not observed in fully undifferentiated ISC populations. Indeed, unlike the results described herein from *Mtgr1*^*−/−*^ crypts, neither loss of *Mtg8* nor *Mtg16* was incompatible with enteroid culture and ISC maintenance ex vivo [15, 71, 72]. Nor could MTG8 or MTG16 functionally compensate for MTGR1 loss, despite the ability of these proteins to form functional heterotetromers [73, 74]. Thus, our data suggests *Mtgr1* is uniquely required among its family members to maintain small intestinal ISC function.

Previous studies have also suggested a role for MTGR1 in ISC biology. Studies to date have implicated MTGR1 in two transcriptional networks known as key regulators of ISC identity and function: Wnt and Notch [3]. Indeed, our previous research has determined that MTGR1 can compete with β-catenin for TCF4 occupancy and, in doing so, suppresses Wnt transcriptional targets in cell lines [18]. MTGR1 can also suppress Notch targets via interactions with CSL, a key Notch effector [17]. Yet despite the ability of MTGR1 to repress ISC-related signaling pathways, which one may expect to augment ISC fitness upon MTGR1 loss, we instead noted that MTGR1 deficiency clearly abrogated ISC function. Thus, further investigation of the function of MTGR1 in ISC-related signaling cascades and identification of bona-fide MTGR1 genomic targets, using methodology such as CUT&RUN and ATAC-seq, will be critical in furthering our understanding of how MTGR1 contributes to crypt and intestinal biology.

Taken together, our data indicates that the failure of *Mtgr1*^*−/−*^ enteroids is a stem-cell intrinsic defect based on decreased ISC-gene expression signatures and a potential shift in cellular respiration to differentiation-associated oxidative phosphorylation. This is further supported by the lack of rescue by Paneth cells alone, instead requiring hyper-activation of ISC-associated signaling to mediate enteroid rescue [68, 69]. However, despite the striking loss of *Mtgr1* null enteroids ex vivo, *Mtgr1*^*−/−*^ ISCs survive in vivo and their intestinal crypts even displayed higher levels of proliferation than WT crypts [14, 17]. It is worth noting that, despite the ISC-intrinsic changes outlined here, this difference in survival may also be due to compensatory signals in the intestinal microenvironment that are lost upon ex vivo culture. As a Wnt producing population, we hypothesized that intestinal stromal cells may be providing these signals [75]; however, co-culture with cultured stroma did not rescue *Mtgr1*^*−/−*^ enteroids, and secretions from *Mtgr1* null fibroblasts stimulate even less Wnt activity than those from WT mice. Further studies are necessary to delineate MTGR1-driven changes to the intestinal microenvironment and how these changes impact ISC biology, particularly as MTGR1 is expressed in multiple intestinal cell types.

It is also notable that the failure and decreased fitness of *Mtgr1*^*−/−*^ enteroid cultures may be representative of defective ISC responses in the setting of intestinal injury. In vivo, *Mtgr1*^*−/−*^ mice are more sensitive to DSS-induced colitis, which demonstrated reduced epithelial proliferation and failed regeneration [16]. MTGR1 likewise appears to be required for the survival of azoxymethane (AOM)-mutated cells in the setting of AOM/DSS-induced inflammatory tumorigenesis; however, clearance of initiated cells was not observed when tumors were initiated via strong Wnt pathway activation (i.e., A*pc*^*1638*^) [76, 77]. Furthermore, perhaps due to chronically deficient *Lgr5* + ISCs, *Mtgr1* null epithelia epithelium appeared to display baseline activation of injury responses. While many CBC and “reserve” ISC markers were decreased or unchanged in *Mtgr1*^*−/−*^ ISCs, expression of *Clusterin* was greatly increased in *Mgr1*^*−/−*^ ISCs, crypts, and enteroids (Supplementary Fig. 8). This facultative “revival stem cell” is induced by a variety of intestinal injuries, including loss of *Lgr5*+ cells [5]. Taken together, it is tempting to speculate that lower overall ISC function induces mild, chronic intestinal injury, and further intestinal injury or ISC perturbation likely overwhelms *Mtgr1* null stem cell faculty.

In conclusion, our studies more fully elucidate the functional contributions of MTGR1 to small intestinal homeostasis and have identified a novel role for MTGR1 in maintaining *Lgr5* + ISC function. While loss of MTGR1 increased *Lgr5* expression and total LGR5+ cell numbers, *Mtgr1*^*−/−*^ ISCs displayed widespread dysregulation of ISC programs and were functionally deficient as compared to WT ISCs, leading to failure of enteroid cultures. Instead, *Mtgr1*^*−/−*^ ISCs lost their proliferative capacity and underwent rapid aberrant differentiation into absorptive enterocytes. Together, these findings indicate that MTGR1 is required for stem cell function in the intestinal epithelium.

### Supplementary information

Supplemental Text

Supplement Figures

Video S1

Video S2

## Data Availability

Raw and processed data for sequencing studies are available from NCBI’s Gene Expression Omnibus and are accessible through GEO Series accession numbers GSE270545 (scRNA-seq) and GSE270546 (bulk RNA-seq). All other data is available from the authors upon request.
