# Supplementary material for: MTGR1 is required to maintain small intestinal stem cell populations
Source: Cell Death Differ. 2024 Jul 25;31(9):1170–83. doi: 10.1038/s41418-024-01346-x (PMC11369156; doi:10.1038/s41418-024-01346-x)
Supplement: Supplementary file 1 — Supplemental Text [file 41418_2024_1346_MOESM1_ESM.docx]

**Supplemental and Extended Methods**

*Enteroid culture*

3D small intestinal organoids, or enteroids, were established from duodenal crypts isolated from 8-12-week old WT and *Mtgr1^-/-^* mice as previously described ((1, 2)). Duodenal crypts were counted and equal numbers plated in growth factor reduced Matrigel® (Corning) and overlaid with “ENR” mini-gut culture media. (Advanced Dulbecco’s modified Eagle’s medium–F-12 [Gibco], 100 U/ml penicillin, 100μg/ml streptomycin, 1X N2 [Gibco], 1X B27 [Gibco], 1X Glutamax [Gibco], 1mM HEPES [Gibco], 20% R-spondin conditioned media [generated from R-spondin-expressing cells generously provided by Dr. Jeff Whitsett, Cincinnati Children’s Hospital], 10% Noggin conditioned media [generated from Noggin-expressing cells generously provided by Dr. G.R. van den Brink, described in (3)], and 50ng/ml EGF [R&D Systems]). Media at plating was supplemented as noted in the text with various inhibitors, described in **Supplemental Table S1**.

*Live cell imaging*

Crypts were plated according to standard protocols and immediately placed in the incubation chamber of the EVOS® FL Auto Cell Imaging System. Enteroids were imaged every 15 minutes over 5 days with periodic location updates to maintain image focus.

*Single cell RNA-sequencing*

*Data collection*

Tissues from mouse jejunum and ileum were used to generate single-cell RNA-seq data, following a methodology similar to previous studies ((4, 5)). In brief, mouse tissues were incubated in a chelating buffer composed of 20mM HEPES and 3mM EDTA in DPBS for 1.25hrs. Tissue was then transferred to 10ml of PBS and shook vigorously for 2-3 minutes to liberate crypts, which were then passed through a 70µm filter and washed by centrifugation at 300*g* for 5 minutes and resuspension in PBS. Isolated crypts were pelleted and resuspended in a solution of cold active protease (5 mg/ml Protease from *Bacillus licheniformis* and 2.5 mg/mL Dnase in PBS) and incubated for 25 minutes at 4°C. Following this, the tissues were gently pipetted 10-20 times to obtain single cells and passed through a 70µm filter into a clean tube. Isolated cells were pelleted by centrifugation at 700g for 5 min and washed in PBS containing 0.02% BSA three times before final resuspension in PBS containing 15% Optiprep and filtered using a 40µm flowmi filter. The resulting cell suspensions underwent filtration, washing, and quality inspection before being loaded onto inDrops for microfluidic capture. The inDrops scRNA-seq procedure was carried out according to a modified protocol (6, 7).

For duodenal samples, mouse tissues were incubated in a chelating buffer composed of 20mM HEPES and 3mM EDTA in DPBS for 1.25hrs. Tissue was then transferred to 10ml of PBS and shook vigorously for 2-3 minutes to liberate crypts, which were then passed through a 70µm filter and washed by centrifugation at 300g for 5 minutes and resuspension in PBS. Isolated crypts were pelleted and resuspended in a solution of cold active protease (5 mg/ml Protease from *Bacillus licheniformis* and 2.5 mg/mL Dnase in PBS) and incubated for 25 minutes at 4°C. Following this, the tissues were gently pipetted 10-20 times to obtain single cells and passed through a 70µm filter into a clean tube. Isolated cells were pelleted by centrifugation at 700g for 5 min and washed in PBS containing 0.02% BSA. Cells were further washed 2 additional times and encapsulated using reagents and consumables in the PIPseq T2 kit (targeting capture of ~2,000 cells) version 4.5 from Fluent Biosciences. For all studies, single-cell libraries were prepared for sequencing as detailed in previous documentation (8, 9). These libraries, each containing an estimated 2000-3000 cell transcriptomes, were then sequenced on the Novaseq6000 platform, generating approximately 125 million reads per library.

*Single cell RNA-sequencing data analysis*

*Data processing*

Data quality was evaluated using ambiQuant (7); data were then filtered using dropkick (10) and further processed according to an established pipeline (11). Briefly, raw scRNA-seq counts were normalized by median library size, log-like transformed with arcsinh, and z-score standardized per gene followed by dimensional reduction and UMAP visualization using Python packages scanpy (12), pandas (13) and numpy (14). Relevant code is available at https://github.com/Ken-Lau-Lab.

*Gene marker and signature comparison*

To compare individual gene and gene signature expression between the wild-type and *Mtgr1* null groups, the two treatment groups underwent random sub-sampling to keep the same number of total cells in each group. Cells of type of interest were extracted, and arcsinh-transformed counts were used for down-stream processes. For individual markers, counts were scaled to the range between 0 and 1. For gene signatures, signature scores were computed using the scanpy.tl.score_genes() function (12)  with a list of genes of interest on arcsinh-transformed counts. Two-sided Mann-Whitney Wilconxon test were performed to evaluate statistical significance with the statannotations Python package (15).

*Cell type composition comparison*

Number of cells for each cell type from the sub-sampled dataset were counted, and their percentage values within each sample were used for a two sample T-test between the wild-type and *Mtgr1* null groups.

*Gene set enrichment analysis*

Differential genes between the wild-type and *Mtgr1* null samples’ stem cell population were computed with the scanpy.tl.rank_genes_groups() function (12) on arcsinh-transformed counts. Differential genes with adjusted p-values <0.05 and log-fold changes >0 were selected as upregulated genes for each treatment group. Enriched pathways were computed with the upregulated gene list using the gseapy.enrichr() function (16) with gene sets in the WikiPathways_2019_Mouse library.

*Transmission electron microscopy*

Specimens were processed for transmission electron microscopy (TEM) and imaged in the Vanderbilt Cell Imaging Shared Resource: Research Electron Microscopy facility according to their established methods. Briefly, enteroid-containing Matrigel plugs were fixed in 2.5% glutaraldehyde in 0.1M cacodylate buffer, pH7.4 at room temperature (RT) for 1 hour, then transferred to 4°C overnight. The samples were washed in 0.1M cacodylate buffer, incubated for 1 hour in 1% osmium tetraoxide at RT, and washed with 0.1M cacodylate buffer. The samples were then dehydrated through a graded ethanol series followed by 3 exchanges of 100% ethanol. Next, the samples were incubated for 5 minutes in 100% ethanol and propylene oxide (PO) followed by 2 exchanges of pure PO. Samples were then infiltrated with 25% Epon 812 resin and 75% PO for 30 minutes at RT. Next, they were infiltrated with Epon 812 resin and PO [1:1] for 1 hour at RT, then overnight at RT. The next day, the samples went through a [3:1] (resin: PO) exchange for 3–4 hours and were incubated with pure epoxy resin overnight. Samples were then incubated in 2 more changes of pure epoxy resin and allowed to polymerize at 60°C for 48 hours.

500–1000 nm-thick sections were cut for ultra-structure identification. Then, 70–80 nm ultra-thin sections were cut from the region of interest, collected on 300-mesh copper grids, and post-stained with 2% uranyl acetate followed by Reynold’s lead citrate. Samples were subsequently imaged on the Philips/FEI Tecnai T12 electron microscope.

*Lgr5+ Isolation and FACS Analysis*

Flow cytometry was performed as previously described (17). Notably, 10 μM ROCK inhibitor (Y-27632) (#12-541-0, Fisher Scientific) was added to all buffers and solutions to maximize cell viability. Briefly, duodena were harvested from *Mtgr1^+/+^* (WT) *Lgr5*-GFP, and *Mtgr1^-/-^ Lgr5*-GFP, and WT (*Lgr5*-GFP-negative, necessary for compensation controls) mice, splayed longitudinally on glass plates on ice, rinsed in ice-cold PBS without calcium or magnesium, and transferred to 50 mL conical tubes containing 15 mL ice-cold epithelial dissociation buffer (3 mM EDTA in PBS without calcium or magnesium). Duodena were incubated in epithelial dissociation buffer with nutation for 15 min at 4 °C, removed from the conical tubes, placed on glass plates on ice, and splayed flat with the luminal epithelial side down (facing the glass). The serosal side was carefully scraped down and back 2x using a pipette tip placed parallel to the tissue to remove villi. Duodena were then minced and incubated in fresh ice-cold epithelial dissociation buffer with nutation for 30 min at 4 °C. Tissue fragments were transferred to 50 mL conical tubes containing 10 mL ice-cold PBS without calcium or magnesium and subjected to 2 min gentle shaking to free crypts. Tissue fragments were allowed to settle, and supernatants were transferred to new tubes and centrifuged at 500 *x g*, 4 °C for 5 min to collect crypts.

Crypt pellets were resuspended in 10 mL pre-warmed Hanks’ Balanced Salt Solution (HBSS) containing 0.3 U/mL dispase (#17105-041, Invitrogen), and 0.1 mg/mL Deoxyribonuclease (DNase) I from bovine pancreas (#DN25-100MG, Sigma-Aldrich) and transferred to new 50 mL conical tubes. Tubes were incubated in a 37 °C bead bath with vigorous shaking every 1-2 min for 30 s up to a total of 10 min. 15-μL aliquots were examined by brightfield microscopy after each shaking cycle. When most crypt epithelial cells had dissociated into singlets or doublets, digestion was halted by filtering the epithelial cell suspension through a 40-μm cell strainer (#352340, Corning) into 10 mL ice-cold PBS with calcium and magnesium containing 10% FBS. Epithelial cells were centrifuged at 500 *x g*, 4 °C for 5 min and washed 3x with 15 mL ice-cold PBS with calcium and magnesium. Cells were counted prior to the final wash. Following the final wash, pelleted cells were resuspended in IntestiCult Organoid Growth Medium (Mouse) (#06005, Stem Cell Technologies) supplemented with 10 μM Y-27632. 1x10^5^ cells were removed for each gating control (Unstained, PI-only, and Annexin V only controls from WT L- cells; GFP-only control from WT L+ cells). If necessary, samples were diluted to a density of 5x10^6^ cells/mL using additional media. Controls and samples were then stained with 1:100 APC Annexin V (#640919 BioLegend) and 1:4000 propidium iodide (PI) (#281487-000, Invitrogen) as appropriate immediately prior to FACS. Samples were FACS-sorted for live, single *Lgr5*-GFPHigh and *Lgr5*-GFPLow cells using the Vanderbilt Flow Cytometry Shared Resource.

*Stromal isolation and co-culture*

Intestinal stromal cells were isolated and cultured from minced intestinal stroma of either WT or *Mtgr1^-/-^* mice according to published methods(18). Briefly, duodenal tissues were minced and crypts were isolated according to standard enteroid culture pipelines. After crypt isolation, tissue fragments were vigorously pipetted and washed to remove remaining epithelial cells. Tissues were next digested for 3 hours at 37^o^ in media containing 1mg/ml Collagenase IV (Sigma) and 1mg/ml Dispase (Roche). After incubation, tissue fragments were pipetted again, strained through a 70um cell strainer, washed, pelleted, and plated in RPMI media (Gibco) containing 10% FBS, 1% Pen/Strep, and 1% Glutamax to generate cultured stroma. Cells were refed after 2 days.

After 5 days, stromal cells were collected and plated with intestinal enteroids at a ratio of 300 crypts to 1.5x10^3^ stromal cells. Viable enteroids were counted daily and normalized to day 1 values, and WT enteroids were further assessed for cystic structures associated with increased Wnt activity (19). Stromal cell conditioned media was also collected and cultured with HEK293 cells expressing the TCF/LEF “Super TOPFlash” reporter construct (20). To detect Wnt-dependent luciferase, cells were lysed in 1× Glo Lysis Buffer (E2661, Promega), and lysates were mixed 1:1 with Steady-Glo luciferase reagent (E2510, Promega) or CellTiter-Glo luminescent cell viability reagent (G7570, Promega) as we have done previously (21). Luminescence was measured with a GloMax Discover microplate reader (Promega), and Steady-Glo readings were normalized to CellTiter-Glo readings to account for any changes in cell viability.

*Rosa^mT/mG^ enteroid isolation and co-culture*

*ROSA^mT/mG^* mice were purchased from The Jackson Laboratory (#007676) and used to establish breeding colonies (22). At 6-8 weeks old, duodenum were collected from *ROSA^mT/mG^* mice and used to establish enteroids. Enteroids were established from *Rosa^mT/mG^* mice, split, and cocultured with an equal number of WT or *Mtgr1^-/-^* crypts. Viable tomato negative enteroids were counted daily and normalized to day 1 values.

**Supplemental Figure Legends**

**Supplemental Figure 1.** **Supplemental scRNA-sequencing.** **(A)** UMAP showing per sample results of Figure 1 scRNA-sequencing experiment (ileum). **(B)** Additional scRNA-sequencing experiment with UMAPs showing cell type, *Mtgr1* (*Cbfa2t2*) expression, and sample distribution in the mouse jejunum.

**Supplemental Figure 2. scRNA-sequencing of WT and *Mtgr1^-/-^* intestine.** **(A)** UMAP of per sample results of Figure 2 scRNA-sequencing experiments from WT and **(B)** *Mtgr1^-/-^* duodenal samples. n=3 WT and 2 *Mtgr1^-/-^* mice. ABS: absorptive, EE: enteroendocrine, GOB: goblet, PAN: Paneth, RevSTM: revival stem, STM: stem, TAC: transit amplifying cell. TUF: tuft.

**Supplemental Figure 3. Changes in cell type distribution and enterocyte differentiation in day 3 *Mtgr1^-/-^* enteroids.** Gene set enrichment analysis (GSEA) of *Mtgr1^-/-^* day 3 enteroid RNA-sequencing results using gene sets representing **(A)** intestinal epithelial cell types and **(B)** microvilli and brush border biology queried from the Gene Ontology collection. NES = normalized enrichment score. Tag % = the percentage of gene hits before (for positive ES) or after (for negative ES) the peak in the running ES, indicating the percentage of genes contributing to the ES. Significance indicated by FDR q value.

**Supplemental Figure 4. Changes in enterocyte-associated genes in the *Mtgr1^-/-^* intestinal crypt.** Gene set enrichment analysis (GSEA) of *Mtgr1^-/-^* crypt RNA-sequencing results using gene sets representing intestinal epithelial cell types. NES = normalized enrichment score. Tag % = the percentage of gene hits before (for positive ES) or after (for negative ES) the peak in the running ES, indicating the percentage of genes contributing to the ES. Significance indicated by FDR q value.

**Supplemental Figure 5. *Mtgr1^-/-^* enteroid survival is not rescued by co-culture with other intestinal cell types.** **(A)** Intestinal stromal cells were isolated from WT and *Mtgr1^-/-^* mice and used to establish *in vitro* stromal cultures. Stromal cells were mixed with crypt isolations in the combinations shown and enteroid viability was quantified daily. **(B)** Quantification of WT enteroids which display a Wnt-associated spheroid phenotype. **(C)** Conditioned media was generated from WT and *Mtgr1^-/-^* stromal cells and cultured overnight with the HEK STF Wnt reporter line. **(D)** Enteroid lines were established from *ROSA^mT/mG^* duodenum and co-cultured with WT and *Mtgr1^-/-^* crypts. Viability was quantified daily. **(E)** Representative images of *ROSA^mT/mG^* co-cultures at day 4 post plating, arrows show plated crypts. n=2 independent experiments with 3-4 technical replicates each. **P*<0.05, ***P*<0.01, ****P*<0.001, *****P*<0.0001, two-way ANOVA (A, D), one-way ANOVA (B), or Student’s t test (C).

**Supplemental Figure 6. ISC-associated gene expression.** **(A)**Distribution of ISC associated genes in WT and **(B)** *Mtgr1^-/^* UMAPS. **(C)** Cell type distribution, also shown in Figure 2. ABS: absorptive, EE: enteroendocrine, GOB: goblet, PAN: Paneth, RevSTM: revival stem, STM: stem, TAC: transit amplifying cell. TUF: tuft.

**Supplemental Figure 7. Total Wikipathways_2019_Mouse GSEA results from ISC populations.** **(A)** All gene sets with an FDR q value>0.05 in WT and **(B)** *Mtgr1^-/-^* ISCs.

**Supplemental Figure 8. Injury-associated ISC programs are increased in the *Mtgr1^-/-^* intestine.** **(A)** Levels of the revival stem cell marker, *Clusterin* (*Clu*), was assessed by fluorescent *in situ* hybridization using RNA Scope technology. Results represent the percentage of crypts containing at least one *Clu*+ cell. **(B)** *Clu* expression was queried in ISC populations from scRNA-seq results. ****P*<0.001, *****P*<0.0001, Student’s t test (A) or Mann-Whitney Wilcoxon test (B).

**Supplemental Video 1.** WT crypts were plated and outgrowth was visualized by live cell imaging over 5 days.

**Supplemental Video 2.** *Mtgr1^-/-^* crypts were plated and outgrowth was visualized by live cell imaging over 5 days.

**Supplemental Tables**

**Supplemental Table S1: Enteroid treatments**

| **Treatment** | **Function** | **Concentration** | **Source** |
| --- | --- | --- | --- |
| DAPT | γ-secretase inhibitor | 10µM, 20µM | Tocris |
| Z-VAD-FMK | Caspase inhibitor | 1µM, 10µM, 100µM | Fisher |
| CHIR99021 | GSK-3 inhibitor | 3µM, 5µM, 10µM | Tocris |
| Necrostatin-1 | RIP1 kinase inhibitor | 10µg/ml, 50µg/ml, 100µg/ml | Cayman Chemical |
| Pifithrin-α | p53 inhibitor | 10µM, 20µM, 20µM | Tocris |

**Supplemental Table S2. qRT-PCR primers**

| **Gene Symbol** | **Forward primer** | **Reverse primer** |
| --- | --- | --- |
| *Lgr5* | CCAATGGAATAAAGACGACGGCAACA | GGGCCTTCAGGTCTTCCTCAAAGTCA |
| *Myc* | ATGCCCCTCAACGTGAACTTC | GTCGCAGATGAAATAGGGCTG |
| *Ki67* | ATCATTGACCGCTCCTTTAGGT | GCTCGCCTTGATGGTTCCT |
| *Ascl2* | AAGCACACCTTGACTGGTACG | AAGTGGACGTTTGCACCTTCA |
| *Olfm4* | TGGCCCTTGGAAGCTGTAGT | ACCTCCTTGGCCATAGCGAA |
| *Muc2* | GGTCCAGGGTCTGGATCACA | GCTCAGCTCACTGCCATCTG |
| *Lyz2* | ATGGAATGGCTGGCTACTATGG | ACCAGTATCGGCTATTGATCTGA |
| *Gapdh* | CCGCATCTTCTTGTGCA | CGGCCAAATCCGTTCA |

**Supplemental Table S3. *P* values for heatmap shown in Figure 5**. *Mtgr1^-/-^* compared to WT. Significantly downregulated and upregulated genes shown by blue and red text, respectively.

|  | **Crypt** | **Day 1** | **Day 3** |
| --- | --- | --- | --- |
| *Lgr5* | 0.322195 | **3.01E-22** | **1.10E-64** |
| *Axin2* | 0.996387 | **2.33E-59** | **6.35E-32** |
| *Ascl2* | 0.820951 | **6.12E-10** | **1.30E-09** |
| *Lrig1* | 0.628468 | **6.01E-29** | **3.57E-25** |
| *Bmi1* | 0.573929 | 0.157114 | 0.704182 |
| *Hopx* | 0.97196 | **1.15E-07** | **0.006656** |
| *Sox9* | 0.912694 | **3.28E-17** | **2.73E-08** |
| *Mki67* | 0.615957 | **7.46E-08** | **1.19E-06** |
| *Cdk1* | 0.914395 | **4.17E-09** | **2.50E-06** |
| *Cdk2* | 0.797865 | **1.31E-09** | **4.09E-06** |
| *Cdk4* | 0.946642 | **5.56E-14** | **5.57E-06** |
| *Cdkn1a* | 0.799407 | **1.90E-08** | **7.56E-15** |
| *Cdkn2b* | 0.168947 | **1.39E-10** | **6.62E-12** |
| *Cdkn1c* | 0.281852 | **6.79E-05** | **0.009381** |

**References**

1. Reddy VK, Short SP, Barrett CW, Mittal MK, Keating CE, Thompson JJ, et al. BVES Regulates Intestinal Stem Cell Programs and Intestinal Crypt Viability after Radiation. Stem Cells. 2016;34(6):1626-36.

2. Parang B, Rosenblatt D, Williams AD, Washington MK, Revetta F, Short SP, et al. The transcriptional corepressor MTGR1 regulates intestinal secretory lineage allocation. FASEB J. 2015;29(3):786-95.

3. Heijmans J, van Lidth de Jeude JF, Koo BK, Rosekrans SL, Wielenga MC, van de Wetering M, et al. ER stress causes rapid loss of intestinal epithelial stemness through activation of the unfolded protein response. Cell Rep. 2013;3(4):1128-39.

4. Banerjee A, Herring CA, Chen B, Kim H, Simmons AJ, Southard-Smith AN, et al. Succinate Produced by Intestinal Microbes Promotes Specification of Tuft Cells to Suppress Ileal Inflammation. Gastroenterology. 2020;159(6):2101-15 e5.

5. Simmons AJ, Lau KS. Dissociation and inDrops microfluidic encapsulation of human gut tissues for single-cell atlasing studies. STAR Protoc. 2022;3(3):101570.

6. Klein AM, Mazutis L, Akartuna I, Tallapragada N, Veres A, Li V, et al. Droplet barcoding for single-cell transcriptomics applied to embryonic stem cells. Cell. 2015;161(5):1187-201.

7. Arceneaux D, Chen Z, Simmons AJ, Heiser CN, Southard-Smith AN, Brenan MJ, et al. A contamination focused approach for optimizing the single-cell RNA-seq experiment. iScience. 2023;26(7):107242.

8. Southard-Smith AN, Simmons AJ, Chen B, Jones AL, Ramirez Solano MA, Vega PN, et al. Dual indexed library design enables compatibility of in-Drop single-cell RNA-sequencing with exAMP chemistry sequencing platforms. BMC Genomics. 2020;21(1):456.

9. Vega PN, Nilsson A, Kumar MP, Niitsu H, Simmons AJ, Ro J, et al. Cancer-Associated Fibroblasts and Squamous Epithelial Cells Constitute a Unique Microenvironment in a Mouse Model of Inflammation-Induced Colon Cancer. Front Oncol. 2022;12:878920.

10. Heiser CN, Wang VM, Chen B, Hughey JJ, Lau KS. Automated quality control and cell identification of droplet-based single-cell data using dropkick. Genome Res. 2021;31(10):1742-52.

11. Chen B, Ramirez-Solano MA, Heiser CN, Liu Q, Lau KS. Processing single-cell RNA-seq data for dimension reduction-based analyses using open-source tools. STAR Protoc. 2021;2(2):100450.

12. Wolf FA, Angerer P, Theis FJ. SCANPY: large-scale single-cell gene expression data analysis. Genome Biol. 2018;19(1):15.

13. McKinney W. Data Structures for Statistical Computing in Python. 9th Python in Science Conference: Proceedings of the 9th Python in Science Conference; 2010. p. 56-61.

14. Harris CR, Millman KJ, van der Walt SJ, Gommers R, Virtanen P, Cournapeau D, et al. Array programming with NumPy. Nature. 2020;585(7825):357-62.

15. Charlier F, Weber, M., Izak, D., Harkin, E., Magnus, M., Lalli, J., et al. Statannotations. 2022.

16. Fang Z, Liu X, Peltz G. GSEApy: a comprehensive package for performing gene set enrichment analysis in Python. Bioinformatics. 2023;39(1).

17. Gracz AD, Puthoff BJ, Magness ST. Identification, isolation, and culture of intestinal epithelial stem cells from murine intestine. Methods Mol Biol. 2012;879:89-107.

18. Kabiri Z, Greicius G, Madan B, Biechele S, Zhong Z, Zaribafzadeh H, et al. Stroma provides an intestinal stem cell niche in the absence of epithelial Wnts. Development. 2014;141(11):2206-15.

19. Dunbar K, Valanciute A, Lima ACS, Vinuela PF, Jamieson T, Rajasekaran V, et al. Aspirin Rescues Wnt-Driven Stem-like Phenotype in Human Intestinal Organoids and Increases the Wnt Antagonist Dickkopf-1. Cell Mol Gastroenterol Hepatol. 2021;11(2):465-89.

20. Xu Q, Wang Y, Dabdoub A, Smallwood PM, Williams J, Woods C, et al. Vascular development in the retina and inner ear: control by Norrin and Frizzled-4, a high-affinity ligand-receptor pair. Cell. 2004;116(6):883-95.

21. Pilat JM, Brown RE, Chen Z, Berle NJ, Othon AP, Washington MK, et al. SELENOP modifies sporadic colorectal carcinogenesis and WNT signaling activity through LRP5/6 interactions. J Clin Invest. 2023;133(13).

22. Muzumdar MD, Tasic B, Miyamichi K, Li L, Luo L. A global double-fluorescent Cre reporter mouse. Genesis. 2007;45(9):593-605.
