# Supplementary material for: MTGR1 is required to maintain small intestinal stem cell populations
Source: Cell Death Differ. 2024 Jul 25;31(9):1170–83. doi: 10.1038/s41418-024-01346-x (PMC11369156; doi:10.1038/s41418-024-01346-x)

Supplemental Data associated with:

MTGR1 is required to maintain small intestinal stem cell populations.

Sarah P. Short, Rachel E. Brown, Zhengyi Chen, Jennifer M. Pilat, Bailey A. McElligott, Leslie M. Meenderink, Alexander C. Bickhard, Koral M. Blunt, Justin Jacobse, Jing Wang, Alan J. Simmons, Yanwen Yu, Yilin Yang, Bobak Parang, Yash A. Choksi, Ken S. Lau, Scott W. Hiebert, and Christopher S. Williams

Supplemental Figure 1

A

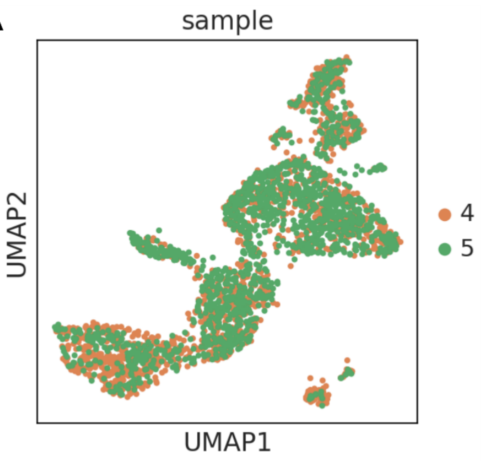

B

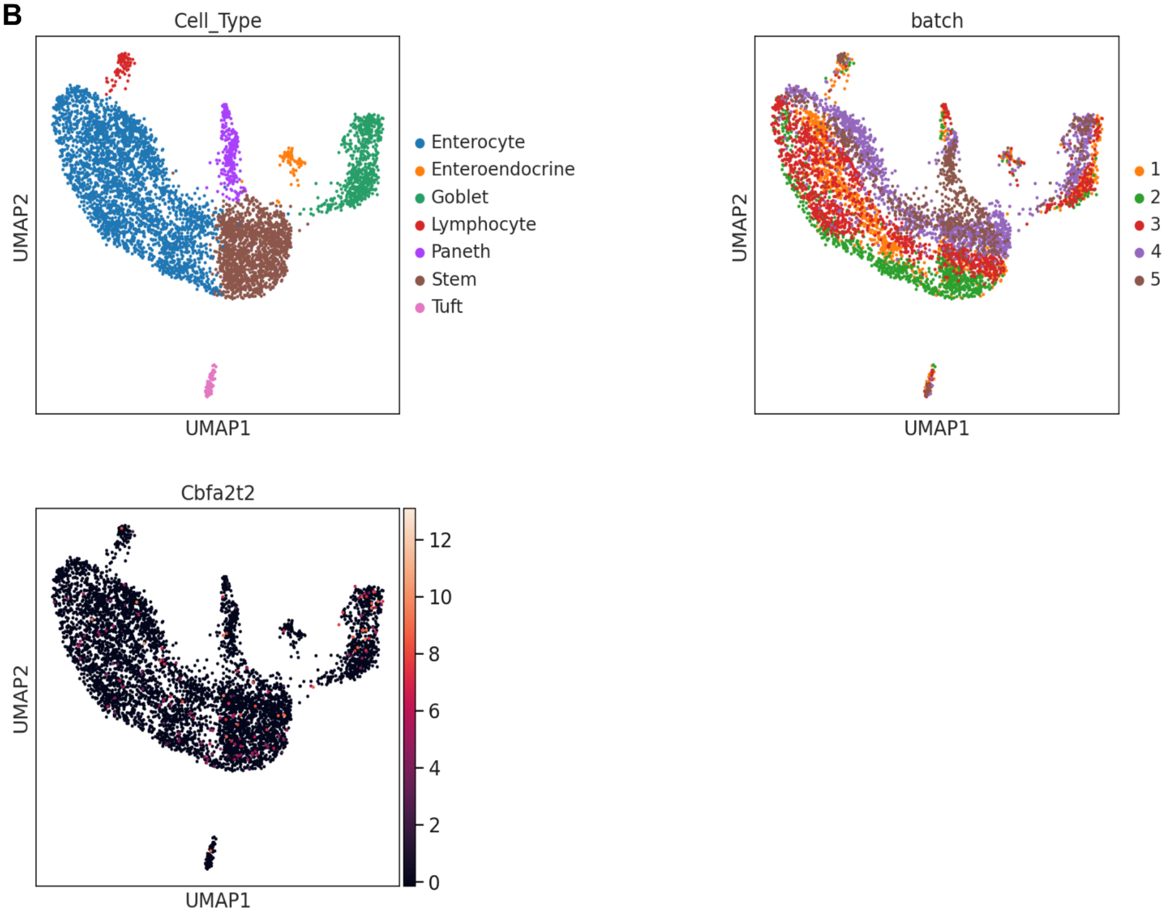

Supplemental Figure 2

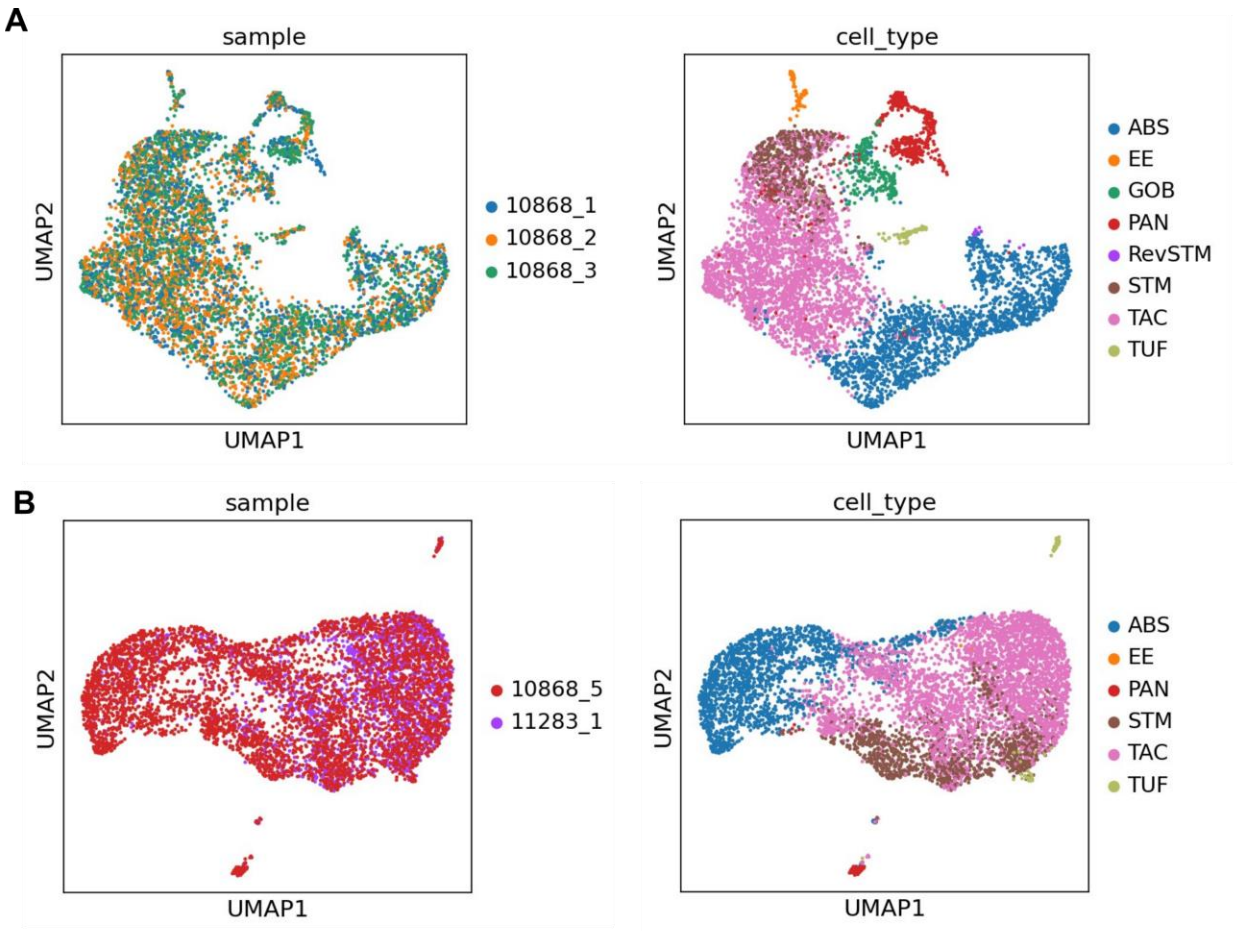

Supplemental Figure 3

A

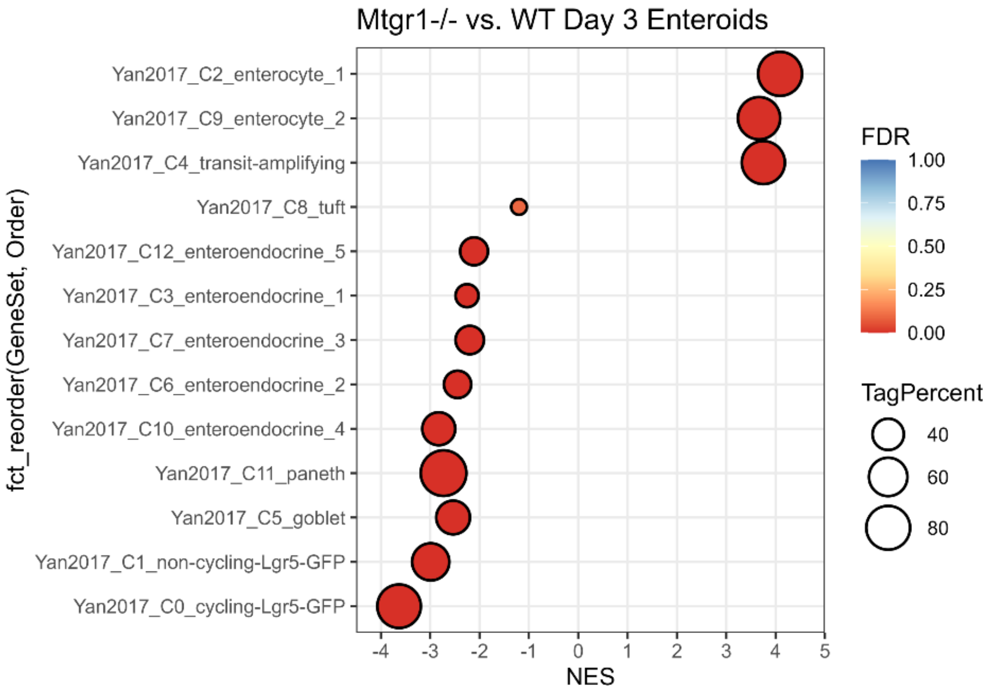

B

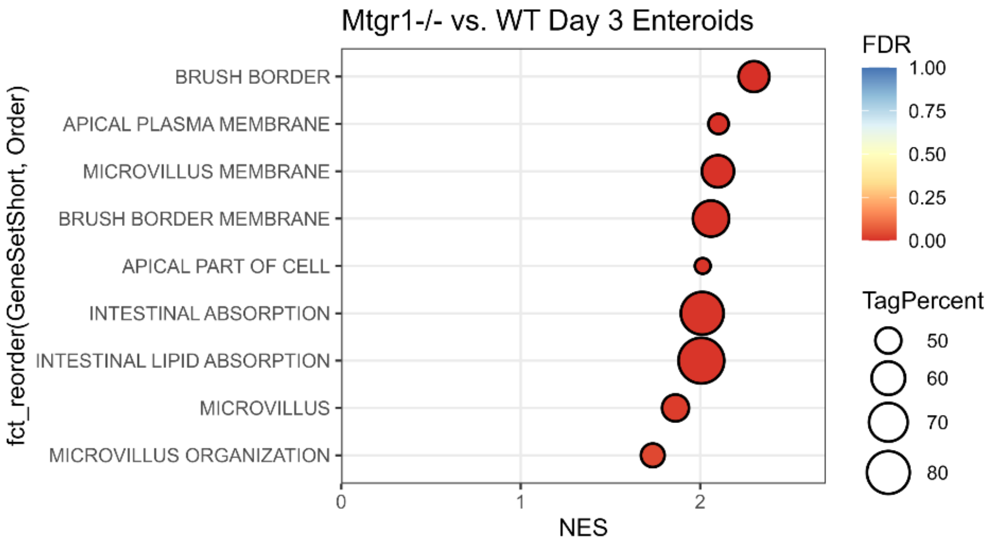

Supplemental Figure 4

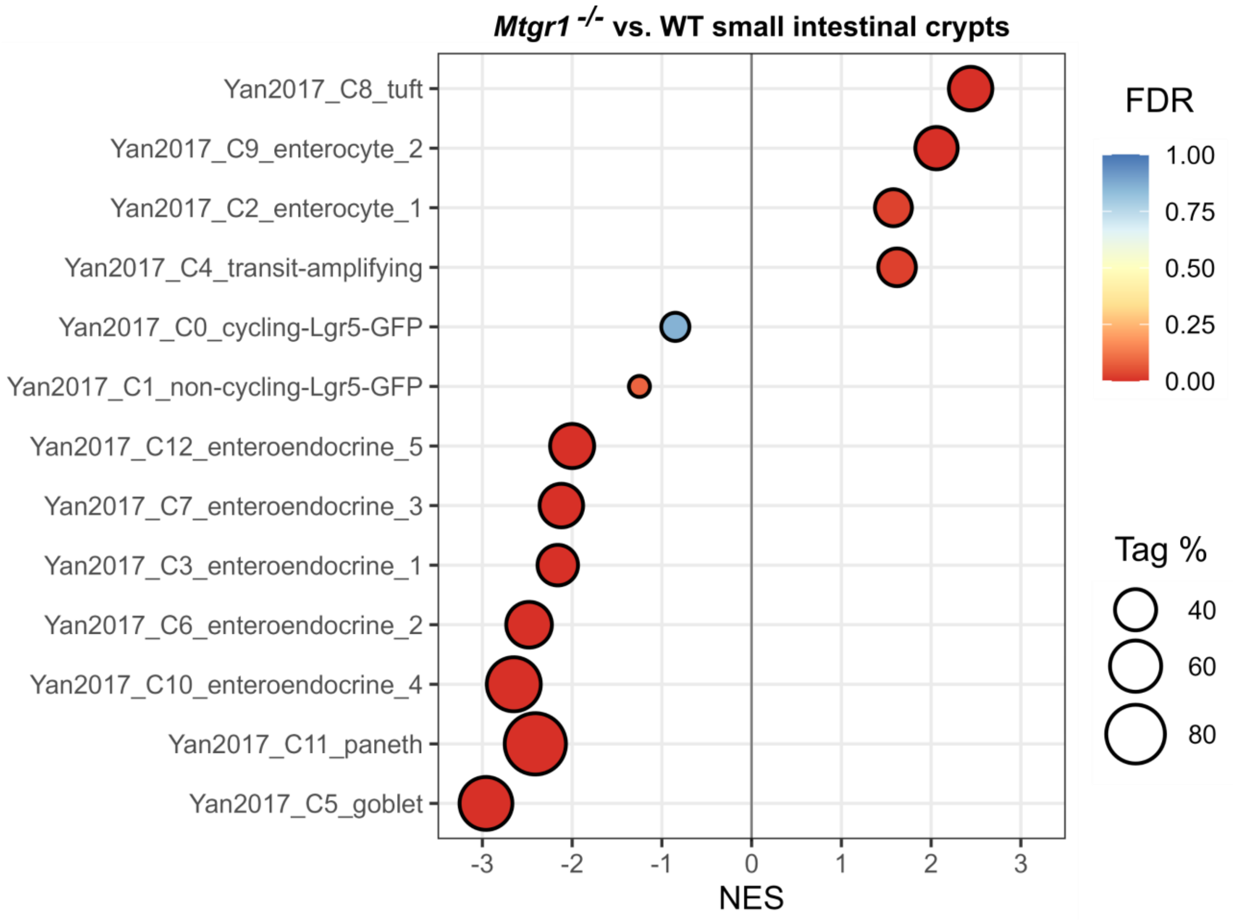

Supplemental Figure 5

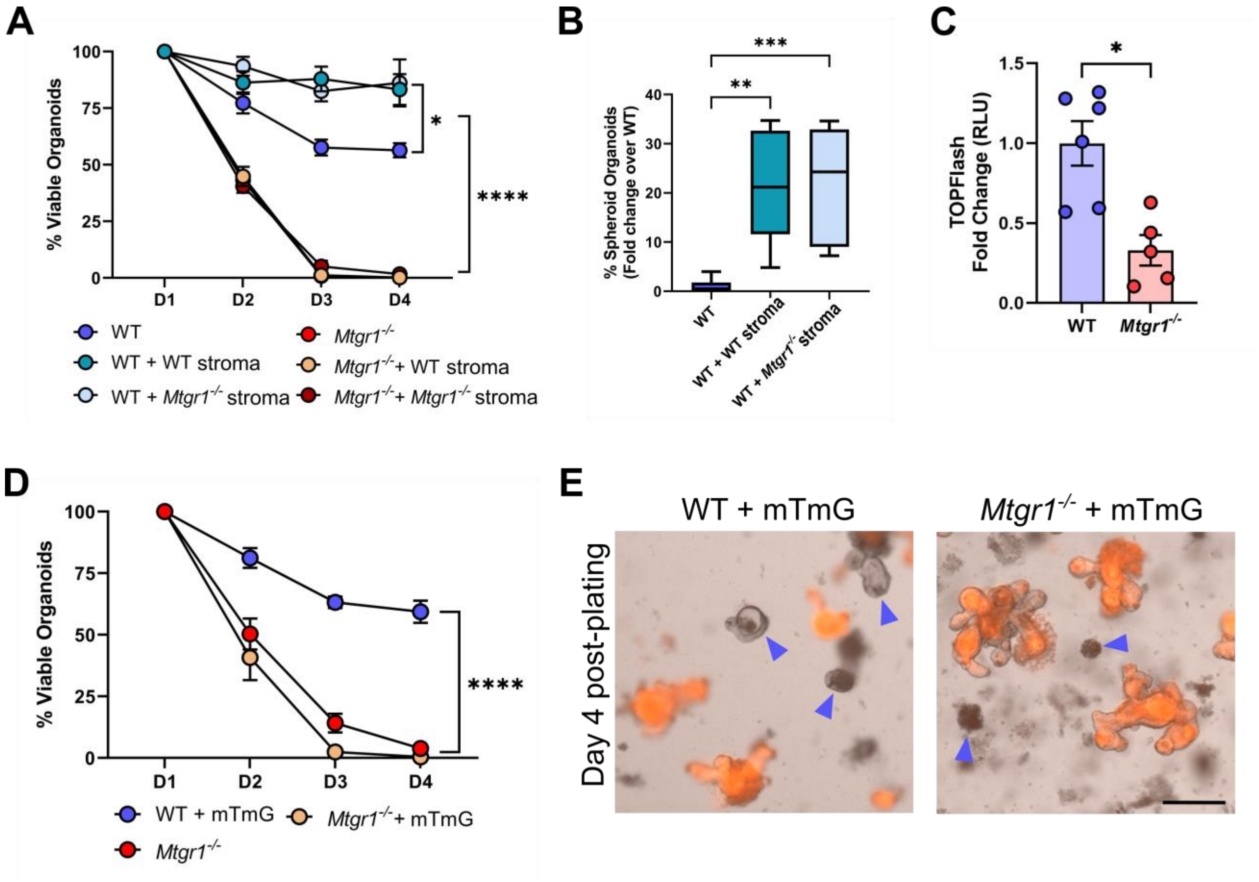

Supplemental Figure 6

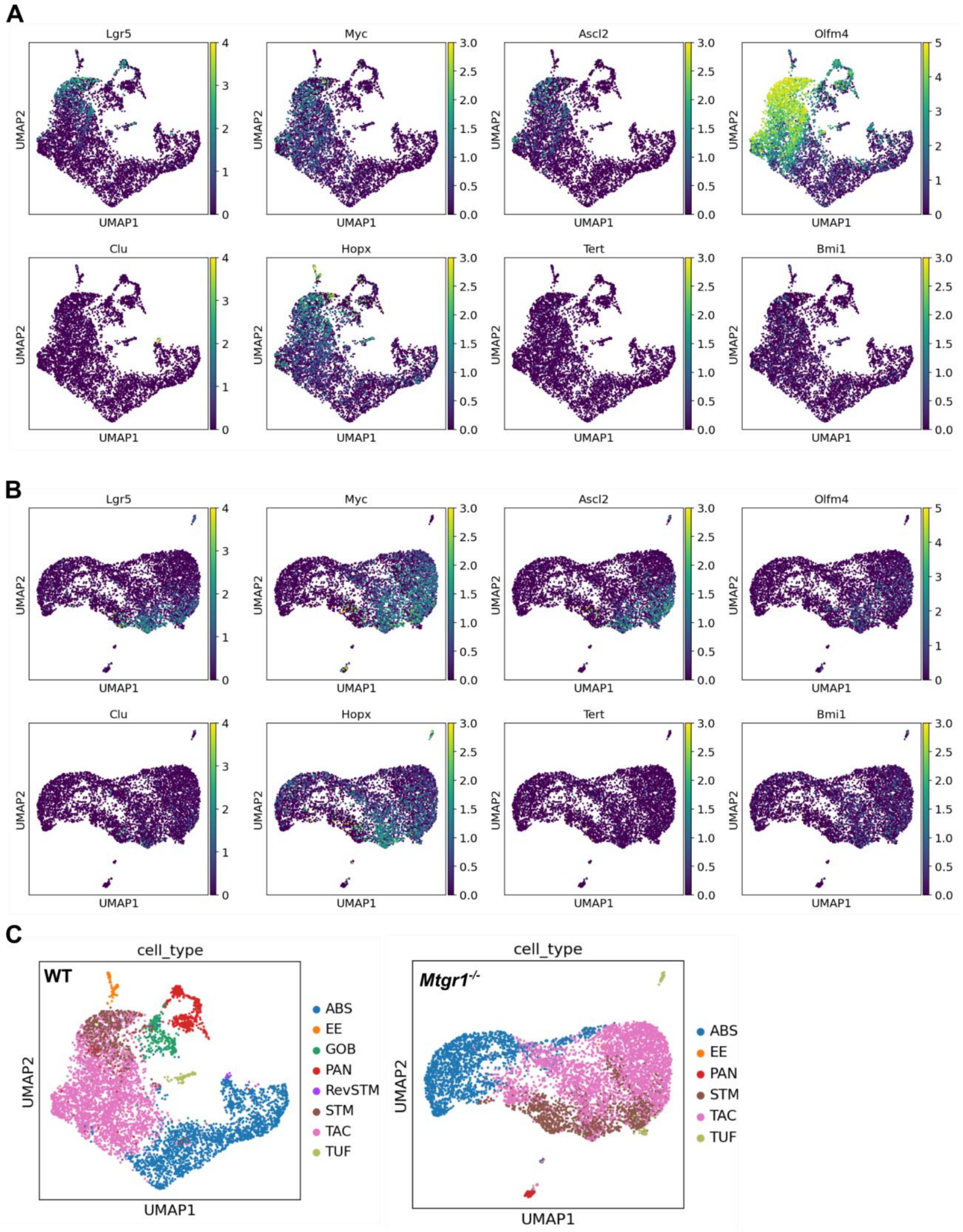

Supplemental Figure 7

A

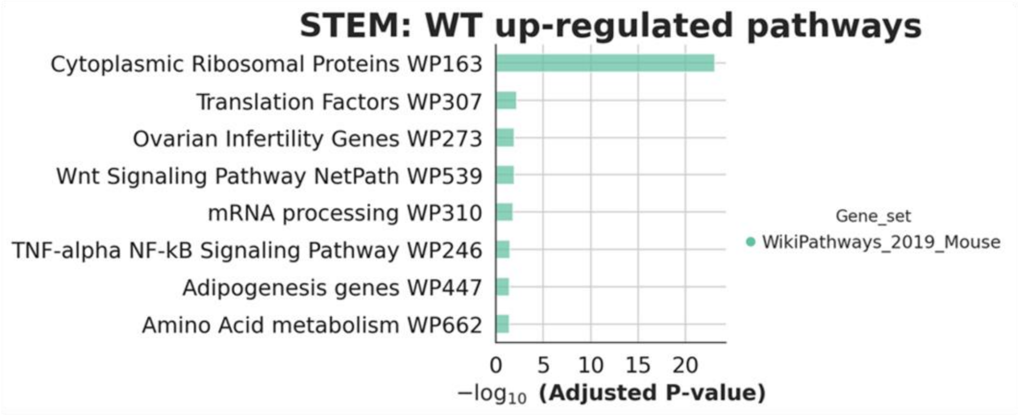

B

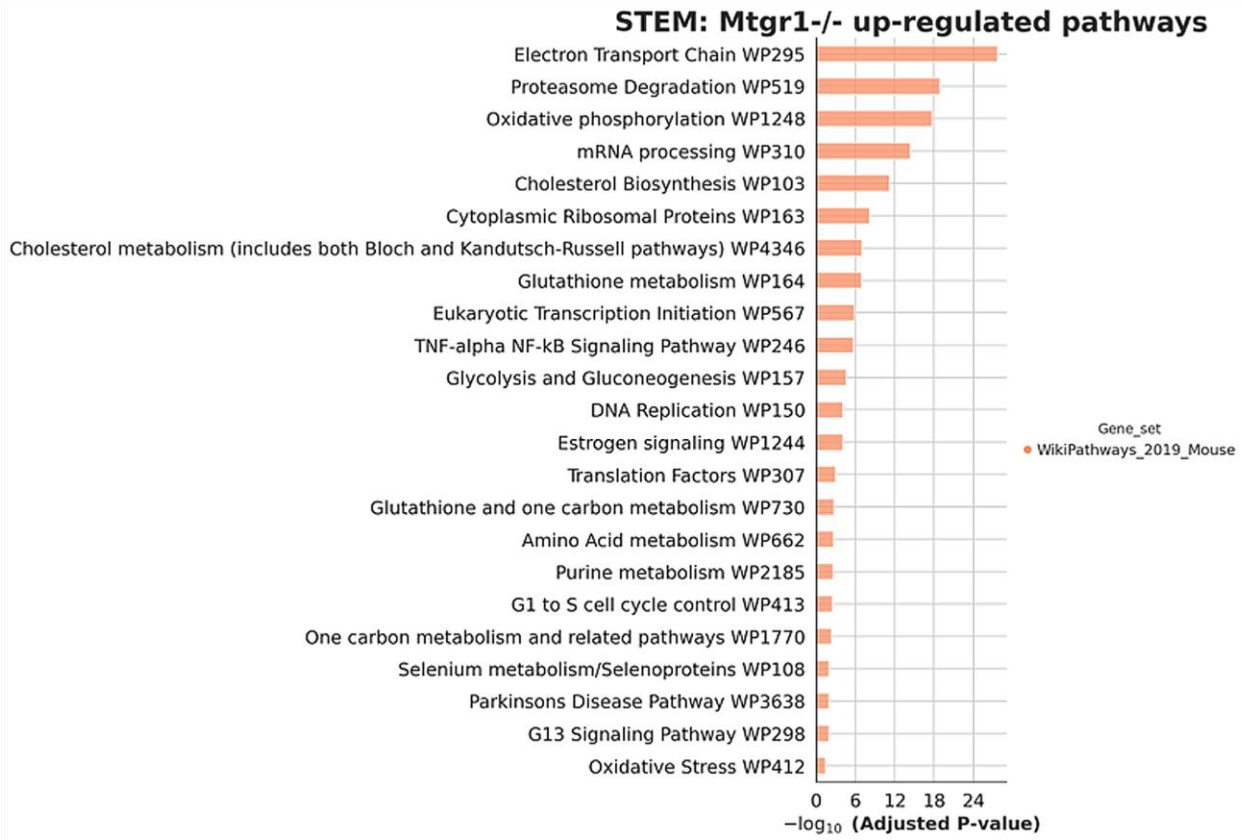

Supplemental Figure 8

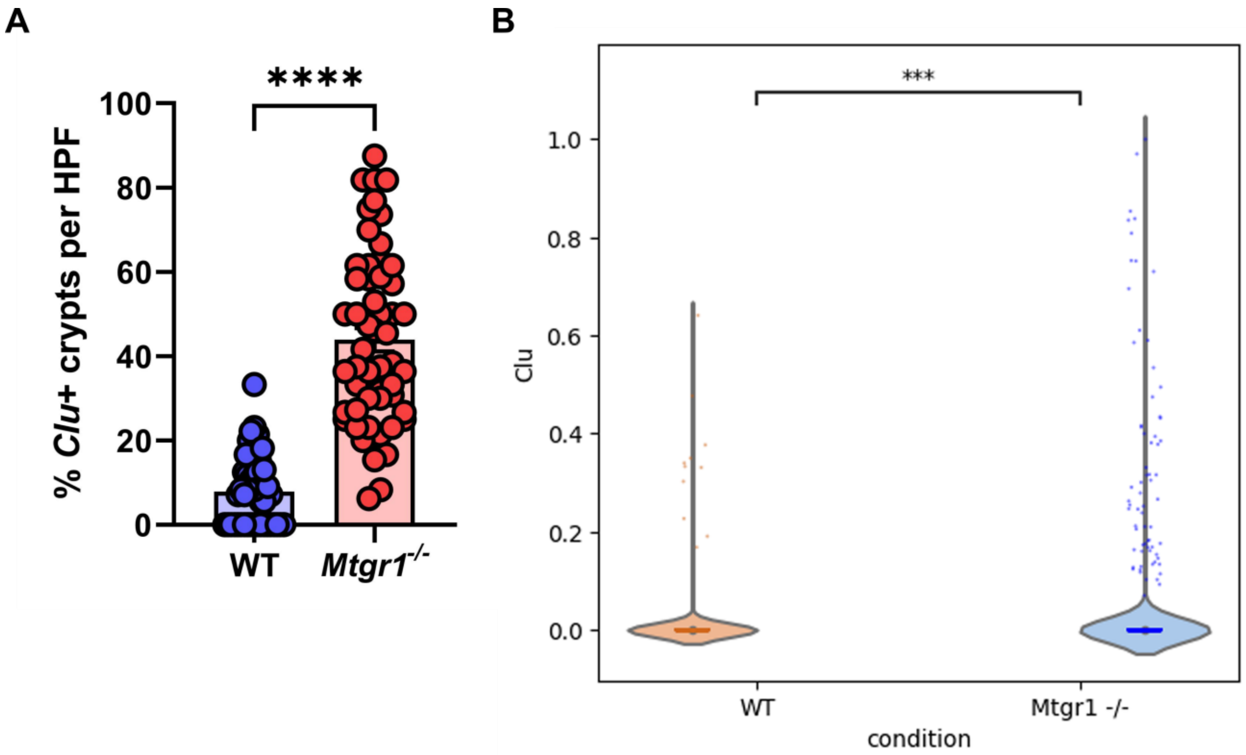

Supplement: Supplementary file 2 — Supplement Figures [file 41418_2024_1346_MOESM2_ESM.pdf]
